# New Insights on the Terpenome of the Red Seaweed *Laurencia dendroidea* (Florideophyceae, Rhodophyta)

**DOI:** 10.3390/md13020879

**Published:** 2015-02-10

**Authors:** Louisi Souza de Oliveira, Diogo Antonio Tschoeke, Aline Santos de Oliveira, Lilian Jorge Hill, Wladimir Costa Paradas, Leonardo Tavares Salgado, Cristiane Carneiro Thompson, Renato Crespo Pereira, Fabiano L. Thompson

**Affiliations:** 1 Institute of Biology, Federal University of Rio de Janeiro (UFRJ) Av. Carlos Chagas Filho, 373-CCS-IB-BLOCO A (ANEXO) A3-202, SAGE-COPPE, Rio de Janeiro 21941-599, Brazil; E-Mails: louisioliveira@gmail.com (L.S.O.); diogoat@gmail.com (D.A.T.); thompsoncristiane@gmail.com (C.C.T.); 2 Federal Institute of Education, Science and Technology of Rio de Janeiro (IFRJ), Campus Duque de Caxias, Avenida República do Paraguai, 120, Sarapuí, Duque de Caxias 25050-100, Brazil; E-Mail: linesoliveira@gmail.com; 3 Research Institute of the Botanical Garden of Rio de Janeiro, Rua Pacheco Leão, 915, Jardim Botânico, Rio de Janeiro 22460-030, Brazil; E-Mails: lilianhill.bio@gmail.com (L.J.H.); wladimirparadas@gmail.com (W.C.P.); lsalgado.jbrj@gmail.com (L.T.S.); 4 Departament of Marine Biology, Federal Fluminense University (UFF), Morro do Valonguinho, s/n, Centro, Niterói 24001-970, Brazil; E-Mail: rcrespo@id.uff.br

**Keywords:** seaweed, terpene synthase, prenyl transferase, gene, secondary metabolite, metabolic pathway, mevalonate pathway, transcriptome, *Laurencia*

## Abstract

The red seaweeds belonging to the genus *Laurencia* are well known as halogenated secondary metabolites producers, mainly terpenoids and acetogennins. Several of these chemicals exhibit important ecological roles and biotechnological applications. However, knowledge regarding the genes involved in the biosynthesis of these compounds is still very limited. We detected 20 different genes involved in the biosynthesis of terpenoid precursors, and 21 different genes coding for terpene synthases that are responsible for the chemical modifications of the terpenoid precursors, resulting in a high diversity of carbon chemical skeletons. In addition, we demonstrate through molecular and cytochemical approaches the occurrence of the mevalonate pathway involved in the biosynthesis of terpenes in *L*. *dendroidea*. This is the first report on terpene synthase genes in seaweeds, enabling further studies on possible heterologous biosynthesis of terpenes from *L*. *dendroidea* exhibiting ecological or biotechnological interest.

## 1. Introduction

Several secondary metabolites produced by marine organisms may interfere in biological interactions at the population, community, and ecosystem levels [1]. Seaweeds commonly produce secondary metabolites to defend themselves against herbivory and biofouling [2] and also to mediate the competitive interactions for space in benthic habitat, acting as allelochemicals [3]. Seaweed species of the genus *Laurencia* are recognized for the biosynthesis of a high diversity of secondary compounds, especially terpenes and acetogennins. This macroalgal genus has been chemically investigated since 1960 [4], but it remains the subject of considerable interest, as evidenced by the recent discovery of new secondary metabolites [5]. The halogenated sesquiterpene (−)-elatol is one interesting example of the major secondary metabolites produced by *Laurencia* species worldwide and it may interact with other compounds to defend this seaweed against herbivory and biofouling [6,7]. Recent studies revealed the variability of (−)-elatol concentration in *L*. *dendroidea* at the intra- and interpopulation levels [8,9], suggesting that this variability is most likely influenced by environmental factors such as temperature and salinity [10].

Some terpenes biosynthesized by *Laurencia* species exhibit a pharmacologically relevant potential due to their strong antiviral [11], antibiotic [12,13], antimalarial [14], antileishmanial [15], antitrypanosomal [16], anti-inflammatory [17] and anti-carcinoma [18,19,20] activities. In addition, the terpenoids from *Laurencia* species have a pronounced anti-epibiosis activity and they could be used for the preparation of antifouling paints [6,7,21]. For example, a first attempt at the commercial application of the sesquiterpene (−)-elatol resulted in a filing of the patent in Brazil to use this compound as an antifouling agent. However, the failure of the large-scale cultivation of *Laurencia* species, the low yield of the extraction process, and the complexity of the organic total synthesis of (−)-elatol in the laboratory [22] are current obstacles to the commercial exploitation of this compound in a biotechnological context. A possible alternative for overcoming this obstacle is the synthesis of terpenes of interest in the laboratory using genetically modified organisms [23]. The molecular engineering of *Escherichia coli* and *Saccharomyces cerevisiae* is a promising alternative, since it allows for the biosynthesis of plant terpenes such as the antimalarial drug artemisinin [24,25], opening up new avenues for the sustainable obtention of terpenoid compounds of biotechnological interest.

In plants and several algae, the biosynthesis of terpenoid precursors can occur through the mevalonate (MVA) pathway and the methylerythritol phosphate (MEP) pathway. In a recent work, we demonstrated the expression of the genes involved in the MEP pathway in *L*. *dendroidea* [26], but there are still no studies reporting the occurrence of the MVA pathway in this species. In fact, the evidence for the simultaneous occurrence of the MEP and MVA pathways in Rhodophyta is currently restricted to biochemical characterizations and gene cloning in *Cyanidium caldarium* and *Galdieria sulphuraria* [27].

The halogenation of organic compounds is a relatively common process in red seaweeds and apparently provides these substances with important features [28,29]. *Laurencia* species biosynthesize a large array of halogenated compounds, which are stored in specialized cellular structures—so-called *corps en cerise* (CC)—in order to avoid autotoxicity [30]. The release of these compounds to the surface of the seaweed is a necessary process to allow their action as defense, and this process occurs in a regulated manner [31]. It depends on the activity of microfilaments that are relevant for the traffic of vesicles containing halogenated metabolites from the CC to the cell periphery [31], and microtubules that participate in the positioning of the vesicles along the cell periphery to specific regions where exocytosis occurs [32]. The frequency of vesicle transport from the CC to the cell surface is influenced by irradiance, desiccation, temperature, and bacterial fouling [33].

Despite the large number of studies analyzing the secondary metabolites, especially terpenes, of *Laurencia* and its chemical, cytological, and ecological aspects, the current knowledge regarding the genes, the biochemical processes, and the cell structures involved in the biosynthesis of these compounds is still limited. In a previous study, we obtained the almost complete set of genes involved in the methylerythritol phosphate (MEP) pathway from *L*. *dendroidea* living under different environmental conditions [26]. In the present study, we expanded our transcriptomic analysis of *L. dendroidea* using clonal axenic cultures under light and dark controlled laboratory conditions and used a cytochemical labeling approach to detect and localize the activity of enzymes involved in the mevalonate (MVA) pathway in this seaweed. Our aim was to analyze the existence of the MVA pathway in *L*. *dendroidea* and unveil the genes related to the biosynthesis of monoterpene, diterpene, triterpene, and sesquiterpene skeletons.

## 2. Results

A total of 5,896,520 sequences were obtained for the transcriptome of *L*. *dendroidea* (Table 1). The relatively large amount of high-quality sequences (with a quality score above 30) obtained from unialgal clones of *L*. *dendroidea*, previously treated with an antibiotic mix and exposed to alternate light and dark conditions, provided an unprecedentedly high coverage of the transcriptome of this seaweed and the possibility to detect new genes associated with the biosynthesis of terpenes. The assembly of all sequences resulted in 54,255 contigs and singlets (Table 1). The genome size of Rhodophyta appears to be highly variable, although the exact values are largely unknown for most species since the complete genome sequence of red macroalgae is currently available only for *Pyropia yezoensis* (43 Mbp) [34] and *Chondrus crispus* (105 Mbp) [35]. The genome size estimate for the species *Laurencia papillosa* is 833 Mbp, based on microspectrophotometry [36]. Taking this value as a reference, and considering that in *Chondrus crispus* around 8% of the genetic material codes for proteins [35], our study would contribute with a 11.5-fold coverage of the transcriptome of *L*. *dendroidea*.

**Table 1:** Characteristics of the sequencing and assembly of the cDNA libraries from the unialgal clones of *Laurencia dendroidea* (SD = Standard deviation).

Sample	Number of Sequences	Total Nucleotides (bp)	Average Size (bp) ± SD
Light	5,825,960	749,118,981	128.0 ± 30
Dark	70,560	15,816,057	224.0 ± 45
Assembled sequences	54,255	19,504,276	359.5 ± 589

**Figure 1:**
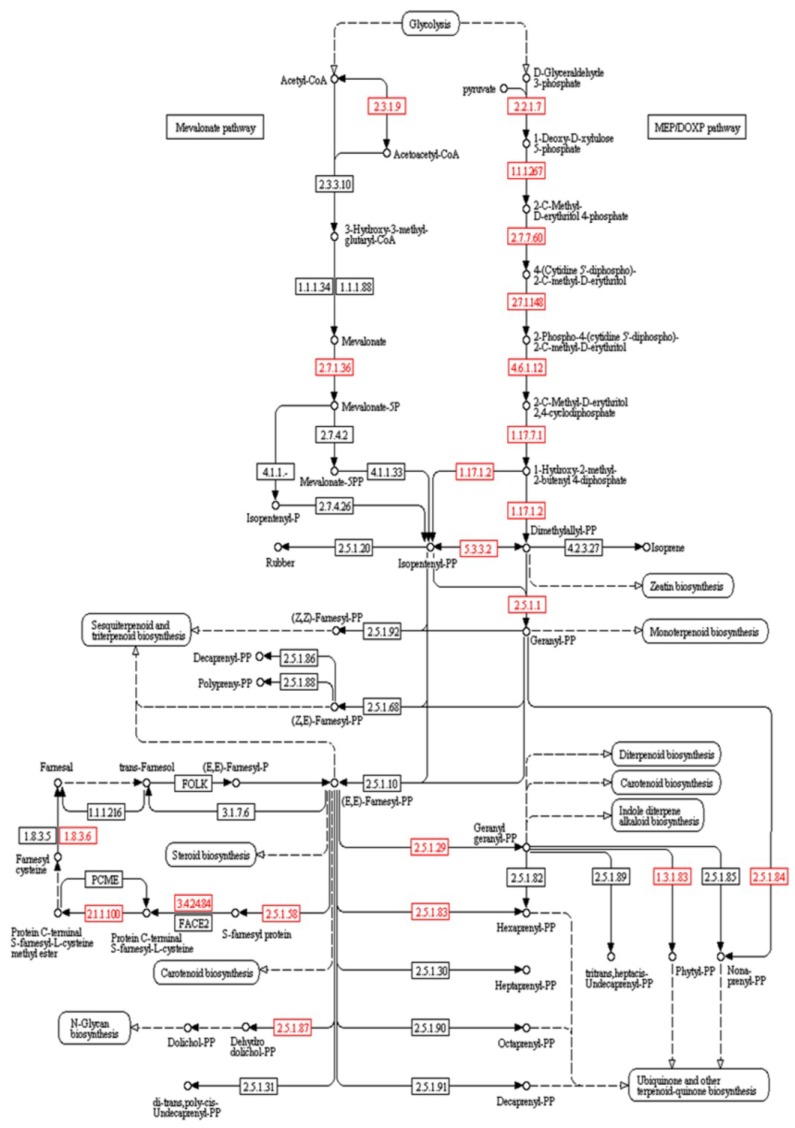
Biosynthetic routes to the terpenoid precursors through the mevalonate (MVA) pathway and the methylerythritol phosphate (MEP) pathway. The red squares represent the genes detected in the transcriptomic analysis of *L*. *dendroidea*. Modified from the Kegg Pathway Database website—Terpenoid backbone biosynthesis reference pathway [37].

The functional annotation of the transcripts revealed for the first time the expression of the genes encoding acetyl-CoA *C*-acetyltransferase (EC 2.3.1.9, Blast e-value: 0.0; similarity: 75%) and mevalonate kinase (EC 2.7.1.36, Blast e-value: 2e-24; similarity: 87%) in *L. dendroidea*, comprising two essential steps for the biosynthesis of isoprenoid precursors through the mevalonate (MVA) pathway (Figure 1).

**Figure 2:**
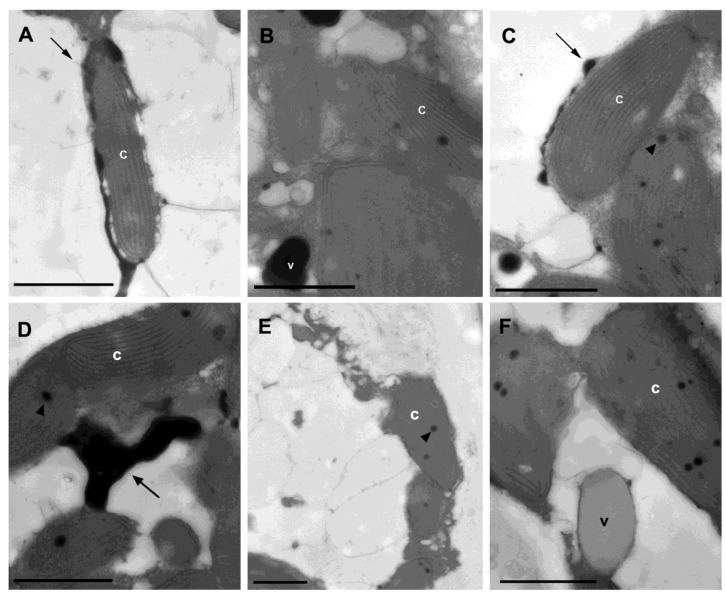
Transmission electron microscopy (TEM) images of *Laurencia dendroidea* spore cells submitted to cytochemical mevalonic acid enzyme assay. (**A**–**D**) indicate the spore cells submitted to the complete acetoacetyl-CoA medium, (**E**) indicates the cells submitted to the substrate control for acetoacetyl-CoA medium, and (**F**) indicates cells submitted to the reaction product control medium. In images (**A**–**D**), arrows are pointing to electron-dense granular material, indicating the HGMS/HGMR activity inside lamellar structures surrounding the chloroplasts (c) (**A**,**C**), in vesicle-like structures (v) near the chloroplasts (**B**), and in large lamellar structures between chloroplasts (**D**). In the control samples (**E**,**F**), no precipitation was verified. The arrowheads indicate plastoglobules. Bars = 1 µm.

Additionally, the activity of 3-hydroxy-3-methylglutaryl-CoA synthase (HMGS, EC 2.3.3.10) and/or 3-hydroxy-3-methylglutaryl-CoA reductase (HMGR, EC 1.1.1.34) was detected through a cytochemical assay. The electron-dense structures indicating the activity of these enzymes were found in spore cells treated with the complete incubation solution containing acetyl-CoA and acetoacetyl-CoA, specifically in lamellar structures surrounding the chloroplasts (Figure 2A,C), in vesicle-like structures near the chloroplasts (Figure 2B), and also in large lamellar structures between chloroplasts (Figure 2D). Electron-dense small spherical structures within the chloroplasts corresponded to the plastoglubules and were present in all samples. The control samples showed no electron-dense deposits (Figure 2E,F). Moreover, we detected the expression of all the genes involved in the methylerythritol phosphate (MEP) pathway (Figure 1). These results indicate the simultaneous occurrence of the MVA and the MEP pathway in *L*. *dendroidea*.

Despite limited knowledge about terpenoid synthase (TPS) in seaweeds, we were able to identify through Blast searches 21 genes from *L. dendroidea* that were similar to well-characterized plant TPS (Table 2 and Supplementary Information). The search for the metal-binding domain (PF03936) and the *N*-terminal domain (PF01397) of terpene synthases did not return new sequences. Still, the search for vanadium-dependent bromoperoxidases returned 11 sequences.

The enzymes involved in the biosynthesis of monoterpenes identified in this study were (3*R*)-linalool synthase (EC 4.2.3.26), (+)-trans-carveol dehydrogenase (EC 1.1.1.275), (+)-pulegone reductase (EC 1.3.1.81), (−)-isopiperitenone reductase (EC 1.3.1.82), and secologanin synthase (EC 1.3.3.9).

**Figure 3:**
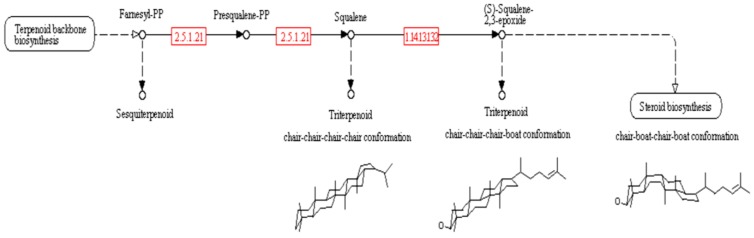
Biosynthetic pathway to the triterpenoid precursors. Modified from the Kegg Pathway Database website—Sesquiterpenoid and triterpenoid biosynthesis reference pathway [65].

The genes coding for farnesyl-diphosphate farnesyltransferase (EC 2.5.1.21) and squalene monooxygenase (EC 1.14.13.132), detected in the transcriptome of *L. dendroidea*, are involved in the biosynthesis of triterpenoid precursors (Figure 3). In this work, we also detected genes involved in the biosynthesis of two different types of triterpenes, represented by the genes coding for squalene-hopene/tetraprenyl-beta-curcumene cyclase (EC 4.2.1.129) and lupeol synthase 1 (EC 5.4.99.41).

Furthermore, the transcriptome of *L. dendroidea* revealed genes associated with the biosynthesis of different classes of sesquiterpenes. The nerolidol synthase gene (EC 4.2.3.48) detected in this transcriptome profile is involved in the biosynthesis of the acyclic sesquiterpene nerolidol; the gene coding for alpha-bisabolene synthase (EC 4.2.3.38) is responsible for the biosynthesis of sesquiterpenes of the bisabolene-type; and the genes coding for germacrene-A synthase (EC 4.2.3.23), germacrene A oxidase (EC 1.1.1.314), aristolochene synthase (EC 4.2.3.9), and 5-epiaristolochene 1,3-dihydroxylase (EC 1.14.13.119) are involved in the biosynthesis of germacrene-type sesquiterpenes. Moreover, we detected the expression of the genes encoding pentalenene synthase (EC 4.2.3.7) and (+)-delta-cadinene synthase (EC 4.2.3.13), which are involved in the biosynthesis of humulene-type and canydil-type sesquiterpenes, respectively. Additionally, the sequence annotation revealed a gene coding for zerumbone synthase (EC 1.1.1.326), which is responsible for the biosynthesis of the sesquiterpene zerumbone.

Finally, three genes involved in the biosynthesis of diterpenes were also detected in the transcriptome of *L. dendroidea*: gibberellin 20-oxidase (EC 1.14.11.12), gibberellin 2-oxidase (EC 1.14.11.13), and abietadienol/abietadienal oxidase (EC 1.14.13.109).

**Table 2:** List of gene names for mono-(C_10_), di-(C_20_), tri-(C_30_), and sesquiterpenes (C_15_) found in the transcriptome of *L*. *dendroidea*, with their products, EC number, Blast e-value and similarity, ecological roles (ER), and biotechnological potential (BP).

Gene Name	EC Number	Blast e-Value	Similarity	Gene Product	Terpene Class	Role	Reference
(3R)-linalool synthase	4.2.3.26	3e-07	68%	Linalool	Monoterpene	Defense (ER); antibiotic, antifungal anticonvulsant, antitumor (BP)	[33,34,35,38,39]
(+)-trans-carveol dehydrogenase	1.1.1.275	2e-08	54%	(+)-(*S*)-carvone	Monoterpene	Anti-herbivore, antifungal (ER); anticonvulsant, antibiotic, cytotoxic, anti-sprouting agent in potatoes (BP).	[40,41,42,43]
(+)-pulegone reductase	1.3.1.81	3e-39	52%	(+)-pulegone	Monoterpene	Defense (ER); analgesic, antibacterial, antifungal, insecticide, acaricidal (BP)	[38,39,44,45,46,47]
(−)-isopiperitenone reductase	1.3.1.82	1e-04	56%	(−)-isopiperitenone (intermediate compound to (−)-menthone)	Monoterpene	Defense (ER); acaricidal, antibiotic (BP)	[38,39,47,48]
secologanin synthase	1.3.3.9	2e-16	52%	Secologanin	Monoterpene	Precursor to indole alkaloids; antimicrobial (BP)	[48,49]
farnesyl-diphosphate farnesyltransferase	2.5.1.21	3e-12	64%	Squalene	Triterpene	Precursor to triterpene	[50]
squalene monooxygenase	1.14.13.132	3e-96	69%	(*S*)-squalene-2,3-epoxide	Triterpene	Precursor to triterpene	[50]
squalene-hopene/tetraprenyl-beta-curcumene cyclase	4.2.1.129	7e-04	62%	hopan-22-ol	Triterpene	Precursor to triterpene with chair-chair-chair-chair conformation	[51]
lupeol synthase 1	5.4.99.41	2e-04	41%	lupeol,β-amyrin	Triterpene	Antibacterial, anti-fungal, anti-inflammatory, antineoplastic, antihypertensive, antiurolithiatic (BP)	[52,53,54]
nerolidol synthase	4.2.3.48	9e-04	62%	Nerolidol	Sesquiterpene	Precursor to α- and β-snyderols	[20,45]
alpha-bisabolene synthase	4.2.3.38	6e-04	43%	(*E*)-alpha-bisabolene	Sesquiterpene	Precursor to (−)-elatol and caespitol; Defense (ER); antileishmanial, anti-trypanosomal, antibiotic, anti-tumor (BP)	[6,7,21,22,26,55,56,57]
germacrene-A synthase	4.2.3.23	6e-20	43%	(+)-(*R*)-gemacrene A	Sesquiterpene	Precursor to germacrene-type sesquiterpenes	[58,59]
germacrene A oxidase	1.1.1.314	3e-9	52%	germacra-1(10),4,11(13)-trien-12-oate	Sesquiterpene	Precursor to germacrene-type sesquiterpenes	[58,59]
aristolochene synthase	4.2.3.9	5e-04	44%	Aristolochene	Sesquiterpene	Precursor to germacrene-type sesquiterpenes	[58,59]
5-epiaristolochene 1,3-dihydroxylase	1.14.13.119	3e-20	53%	Capsidiol	Sesquiterpene	Plant defense (ER); Antibiotic, prostaglandin inhibitor (BP)	[52,60,61,62]
pentalenene synthase	4.2.3.7	1e-07	42%	Pentalenene	Sesquiterpene	Precursor to humulene-type sesquiterpene	[63]
(+)-delta-cadinene synthase	4.2.3.13	3e-05	48%	Precursor to (−)-δ-cadinene and (+)-α-cadinol	Sesquiterpene	Plant defense (ER), antibiotic (BP)	[60,61]
zerumbone synthase	1.1.1.326	5e-08	43%	Zerumbone	Sesquiterpene	Antitumor, anti Alzheimer’s disease (BP)	[52,62]
gibberellin 20-oxidase	1.14.11.12	2e-12	43%	gibberellin 44	Diterpene	Endogenous growth regulators (ER)	[53,54]
gibberellin 2-oxidase	1.14.11.13	7e-11	45%	2beta-hydroxygibberellin 1	Diterpene	Endogenous growth regulators (ER)	[53,54]
abietadienol/abietadienal oxidase	1.14.13.109	1e-13	46%	diterpene acids	Diterpene	Intermediate to diverse diterpene skeletons	[64]

## 3. Discussion

The class of compounds known as terpenes includes some primary metabolites, such as sterols and carotenes; they are part of the molecules of chlorophylls (with a C_20_ isoprenoid side-chain) and of plastoquinone, phylloquinone, and ubiquinone (with long isoprenoid side-chains), which are essential for the survival of the producing organisms [49]. Nevertheless, most of the terpenes found in plants are classified as secondary metabolites, acting mainly as defensive compounds. More than 30,000 terpenoid compounds have been identified to date, all of them derived from diverse combinations of dimethylallyl pyrophosphate (DMAPP) and isopentenyl pyrophosphate (IPP) by the activity of prenyl transferases, resulting in the biosynthesis of monoterpenoids (C_10_), sesquiterpenoids (C_15_), diterpenoids (C_20_), and triterpenoids (C_30_). In this work we detected 20 different genes involved in the biosynthesis of these terpenoid precursors.

The transcriptomic analysis of *L. dendroidea* revealed for the first time the expression of the genes encoding acetyl-CoA C-acetyltransferase (EC 2.3.1.9) and mevalonate kinase (EC 2.7.1.36), which are involved in the biosynthesis of isoprenoid precursors through the mevalonate (MVA) pathway. Furthermore, in the cytochemical assay, some spherical (vesicle-like) and lamellar intracellular structures of *L*. *dendroidea* spores were positively labeled, indicating the activity of 3-hydroxy-3-methylglutaryl-CoA synthase (HMGS, EC 2.3.3.10) and/or 3-hydroxy-3-methylglutaryl-CoA reductase (HMGR, EC 1.1.1.34), and thus supporting the occurrence of the MVA pathway in *L*. *dendroidea*.

Two different and nonhomologous pathways are known to produce isopentenyl pyrophosphate (IPP) and dimethylallyl pyrophosphate (DMAPP), the central intermediates in the biosynthesis of isoprenoids: the mevalonate (MVA) pathway in some eukaryotes and archaea, and the methylerythritol phosphate (MEP) pathway in bacteria and several photosynthetic eukaryotes. In the MVA pathway, the acetyl-coenzyme A is converted to IPP through mevalonate. In *L*. *dendroidea*, the main sites presenting the activity of the enzymes from the mevalonate pathway HMGS and/or HMGR were vesicle-like structures and well-defined lamellar structures. Similarly, HMGR-enriched vesicles were observed in *Arabipdosi*s cells [66], which might have characteristic enzymatic composition specialized in the biosynthesis of isoprenoids. Furthermore, a major HMGR activity was detected in microsomal fractions in *Pisum sativum* seedlings, possibly corresponding to Golgi apparatus and membrane profiles [67]. In addition, we observed HMGS/HMGR activity surrounding the chloroplasts in lamellar inclusions not delimited by membrane, indicating an initial step of the synthesis occurring in the cytoplasm (Figure 2). Likewise, the HMG-CoA reductase was also localized outside the chloroplasts in *Nepeta cataria* leaves [68]. The labeling of regions near the chloroplasts can be related to a role of this organelle in the mevalonate pathway, since the chloroplasts are known to produce acetyl-CoA [69], the first substrate used in the mevalonate dependent pathway. Thus, considering a possible initial step in the cytoplasm, these findings suggest a posterior multi-organelle compartmentalization of the mevalonate pathway, which can be an important intracellular channeling mechanism for the biosynthesis of the different classes of terpenes. Several hypotheses regarding the evolutionary origin of these metabolic pathways have been put forward in the past years, but a recent study suggests that the MVA pathway was probably present in the last common ancestor of all organisms [70], and secondarily lost in some evolutionary lineages, such as the green algae (Chlorophyta) [71].

In addition, we detected all the genes involved in the methylerythritol phosphate (MEP) pathway in *L*.* dendroidea*. The MEP pathway, present in cyanobacteria, was probably transferred to photosynthetic eukaryotes through endosymbiotic events [71]. Plants and red algae retained the ability to biosynthesize terpenoid precursors through both the MVA and the MEP pathways [27]. In general, the MVA pathway is involved in the biosynthesis of sterols, triterpenes, and some sesquiterpenes, while the MEP pathway provides the precursors to monoterpenes, diterpenes, certain sesquiterpenes, carotenoids, and the side chains of chlorophyll and plastoquinone [72]. However, several terpenes have a mixed origin, suggesting some level of interaction between these pathways [73]. In this context, a cross-talk mechanism is relevant to regulate the IPP/DMAPP supply according to the cell demands.

The huge diversity of terpenoid compounds stems from the skeleton modifications catalyzed by terpene synthases (TPS), a mid-size family, with variable gene numbers in sequenced plant genomes [74] and basically uncharacterized in seaweeds. The skeletal diversity arises from the number of terpene synthases, but also from the ability of a single TPS enzyme to generate multiple products using a single substrate, because of the stochastic nature of bond rearrangements that follow the creation of the carbocation intermediates, which undergo a series of cyclizations, isomerizations, hydride shifts, methyl shifts, or other rearrangements [75]. 

Species of the genus *Laurencia* are recognized as the most prolific source of terpenes in the marine environment: They biosynthesize diverse monoterpenes, diterpenes, sesquiterpenes, and triterpenes with relevant ecological and biotechnological potential [50]. Notwithstanding the high number of studies in the past 50 years regarding the discovery and chemical characterization of new terpenoid compounds from *Laurencia* species [4,5], this is the first survey of the genes coding terpene synthases in this genus. In fact, to our knowledge this is the first molecular study addressing this relevant family of enzymes in seaweeds. In this work we report the occurrence of 21 terpene synthases in *L*. *dendroidea* based on Hidden Markov Models profiles and sequence similarity to genes previously described, mainly in plants. The blast e-values for all these genes were equal to or below e-04, and the similarity with the corresponding genes in the available databases was above 40%, conferring reliability to the sequence annotation (Table 2) [51]. Additionally, we provide in the Supplementary File a comparison between the domain composition of these sequences from *L*. *dendroidea* and the corresponding reference genes available at SwissProt/UniProt and PlantCycDB databases.

We also detected the expression of the gene-encoding, vanadium-dependent bromoperoxidase. This enzyme is involved in the halogenation and cyclization of terpenes in red seaweeds [62], and is probably related to the chemical defense of Rhodophyta in response to infection signals [76]. Taking into account the high evolutionary divergence between plants and seaweeds and the ecological and biotechnological relevance of the algal terpenes, this study provides essential information and points to the need to extend molecular research on seaweeds, especially concerning genes of ecological and biotechnological interest associated with secondary metabolism.

### 3.1. Monoterpene Synthase Genes

The expression of the monoterpene synthase genes (3*R*)-linalool synthase (EC 4.2.3.26), (+)-trans-carveol dehydrogenase (EC 1.1.1.275), (+)-pulegone reductase (EC 1.3.1.81), (−)-isopiperitenone reductase (EC 1.3.1.82), and secologanin synthase (EC 1.3.3.9) was detected in the transcriptome of *L*. *dendroidea* (Table 2). The linalool is an acyclic monoterpene possibly involved in signaling pathways related to defense, since the expression of (3*R*)-linalool synthase in plants is induced by wounding and jasmonic acid (JA) [42], and linalool-accumulating transgenic rice plants show an upregulation of defense-related genes [43]. The monoterpene linalool was detected in the red seaweed *Portieria hornemannii* by GC-MS [77], but its role in algae has not yet been demonstrated. Considering that the treatment of the seaweeds *Fucus vesiculosus* and *Chondrus crispus* with methyl jasmonate results in an increase in the biosynthesis of defense compounds and in the transcription of stress-related genes [78,79], we could propose a role for linalool in seaweeds as a signal molecule involved in defense, similarly to that observed in plants, although further studies are necessary to test this hypothesis. This metabolite also displays a pharmacological potential due to its antibiotic [80], antifungal [40], anticonvulsant [41], and antitumor [58] activities.

The enzyme (+)-trans-carveol dehydrogenase is responsible for the conversion of (+)-trans-carveol to (+)-(*S*)-carvone. This secondary metabolite acts as a feeding deterrent and antifungal compound in plants [46,47], and was also detected in *P*. *hornemannii* by GC-MS [77], despite the missing information about its role in seaweeds. Some biotechnologically interesting activities of (+)-(*S*)-carvone include its anticonvulsant [44], antibiotic, and cytotoxic roles; it also acts as an anti-sprouting agent in potatoes [45].

The enzymes (+)-pulegone reductase and (−)-isopiperitenone reductase are involved in the biosynthesis of (+)-pulegone and (−)-menthone, which act as defense compounds in plants [81,82]. In addition, (−)-menthone has acaricidal [83] and antibiotic activities [84], and (+)-pulegone presents analgesic [38], antibacterial, antifungal [39] insecticidal [48], and acaricidal activities [83]. Despite the high similarity of some sequences from *L. dendroidea* with these plant genes (e-value up to e-39), the enzymes and their metabolic products were not previously detected in seaweeds, possibly suggesting the presence of homologous genes in *L. dendroidea* that could be responsible for a similar reaction in this seaweed, since these enzymes act as monoterpene double-bond reductases.

The gene for secologanin synthase, identified in this transcriptome, participates in the biosynthesis of secologanin, a precursor for the production of indole alkaloids. In the marine environment, most of the indole group alkaloids are concentrated in red seaweeds [59]. Several brominated indoles were previously isolated from *Laurencia* *brongniartii* [85], *L. decumbens*, and *L*. *similis* [86], some of them with an antimicrobial activity.

### 3.2. Triterpene Synthase Genes

The genus *Laurencia* is a prolific source of secondary metabolites derived from squalene [87], some of them with a relevant pharmacological activity, such as cytotoxicity against cancer cell lines [88]. The genes coding farnesyl-diphosphate farnesyltransferase (EC 2.5.1.21) and squalene monooxygenase (EC 1.14.13.132), encountered in the transcriptome of *L. dendroidea* are essential to the biosynthesis of triterpenoid precursors [55] (Figure 3). Moreover, we detected genes involved in the biosynthesis of two different types of triterpenes in the transcriptome of *L*. *dendroidea*. The gene encoding squalene-hopene/tetraprenyl-beta-curcumene cyclase (EC 4.2.1.129) is involved in the biosynthesis of triterpenes with a chair-chair-chair-chair conformation [56], and the gene coding lupeol synthase 1 (EC 5.4.99.41) is related to the biosynthesis of triterpenes with a chair-chair-chair-boat conformation. The enzyme lupeol synthase 1 is involved in the biosynthesis of pentacyclic triterpenes, and in *Arabidopsis thaliana* it catalyzes the production of not only lupeol, but some other metabolites, such as β-amyrin. Some squalene-derived pentacyclic triterpenes were previously isolated from *Laurencia* species [89], including the β-amyrin, which is known for its medically relevant antibacterial [57], anti-fungal, and anti-inflammatory activities [60]. Further, lupeol displays antineoplastic, anti-inflammatory, antihypertensive, and antiurolithiatic activities [61].

### 3.3. Sesquiterpene Synthase Genes

**Figure 4:**
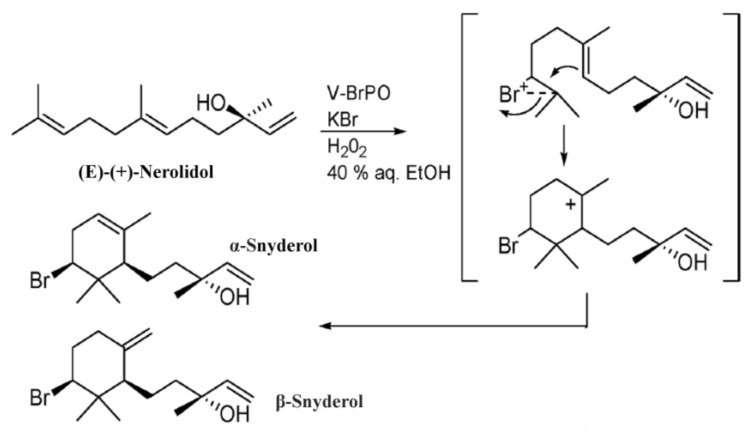
Proposed mechanism for the biosynthesis of α- and β-snyderols from (*E*)-(+)-nerolidol (adapted from [62]).

The nerolidol synthase gene (EC 4.2.3.48) detected in this transcriptome profile is responsible for the biosynthesis of nerolidol, an acyclic sesquiterpene that through a bromonium-ion-induced cyclization by vanadium-dependent bromoperoxidase, generates α- and β-snyderols [62], secondary metabolites isolated from *L*. *obtusa* [14] (Figure 4). Moreover, nerolidol presents antileishmanial [90], antischistosomal [91], and antiulcer [92] activities.

Moreover, we detected the gene coding alpha-bisabolene synthase (EC 4.2.3.38), which is responsible for the biosynthesis of sesquiterpenes of the bisabolene-type. The biosynthesis of (−)-elatol, one of the major secondary metabolites from L. dendroidea, and of some other relevant sesquiterpenes is suggested to involve the enzymatic addition of bromochloride at a bisaboloniun ion stage before the cyclization to the chamigrene derivative [93]. In addition, some other bisabolene-type sesquiterpenes, such as caespitol, filiformin, and β-bisabolene sesquiterpenoids, were previously reported for the genus Laurencia [64,94,95]. The sesquiterpene (−)-elatol plays important ecological roles defending Laurencia against biofoulers [7] herbivores [6], and marine pathogenic bacteria [52]. In addition, this compound has important pharmacological potential due to its antileishmanial [15], anti-trypanosomal [16], antibiotic [53], and anti-tumor effects [54]. Also, caespitol displays a relevant cytotoxic activity against three human tumor cell lines [20].

The enzymes germacrene-A synthase (EC 4.2.3.23), germacrene A oxidase (EC 1.1.1.314), aristolochene synthase (EC 4.2.3.9), and 5-epiaristolochene 1,3-dihydroxylase (EC 1.14.13.119) are involved in the biosynthesis of germacrene-type sesquiterpenes [63,96]. Germacrene is the biosynthetic precursor of the eudesmane class and some other secondary metabolites previously isolated from *L*. *microcladia* and *L*. *filiformis* [97,98,99]. The 5-epiaristolochene 1,3-dihydroxylase is responsible for the biosynthesis of capsidiol, which is involved in plant defense [100,101], and has a pharmacological potential derived from its antibiotic [102] and prostaglandin inhibition activities [103]. 

Moreover, we detected the expression of the genes coding pentalenene synthase (EC 4.2.3.7) and (+)-delta-cadinene synthase (EC 4.2.3.13), which are involved in the biosynthesis of humulene-type and canydil-type sesquiterpenes, respectively. There are few reports on the occurrence of sesquiterpenes from these skeletal classes in *Laurencia*, except for dactylol, previously isolated from *Laurencia poitei*, which is assumed to be biosynthetically derived from humulene [104] and (−)-δ-cadinene and (+)-α-cadinol, which are canydil-type sesquiterpenes isolated from *L*. *microcladia* [98]. Despite the few reports of canydil-type sesquiterpenes in seaweeds, including *Laurencia* and some other red and brown seaweeds [77,105], these types of metabolites are widespread in terrestrial vascular plants. The fungal-elicited production of δ-cadinene synthase in cotton suggests a role for this enzyme in plant defense [106], and the antibiotic activity against *Streptococcus pneumoniae* resistant to conventional antibiotics imply a pharmacological potential for δ-cadinene [107].

Additionally, the sequence annotation revealed a gene coding zerumbone synthase (EC 1.1.1.326), which is related to the biosynthesis of zerumbone, a plant humulane sesquiterpenoid with antitumor activity [108] and a potential candidate for the developmental of anti-Alzheimer’s disease treatment [109]. There is no report on the occurrence of this secondary metabolite in seaweeds. However, our findings possibly indicate the existence of a homologous gene in *L. dendroidea* that could be involved in a similar chemical reaction.

### 3.4. Diterpene Synthase Genes

*Laurencia* species biosynthesize diterpenoid compounds that can be involved in the defense of these seaweeds [110] and also show some pharmacologically relevant antibiotic [111], cytotoxic [112], and anti-inflammatory activities [17]. Through the analysis of the transcriptome of *L*. *dendroidea*, we detected the expression of three genes involved in the biosynthesis of diterpenes: the gibberellin 20-oxidase (EC 1.14.11.12), the gibberellin 2-oxidase (EC 1.14.11.13), and the abietadienol/abietadienal oxidase genes (EC 1.14.13.109) (Table 2 and Supplementary Material). Notwithstanding the absence of information regarding the biosynthesis of abietic acid and derivatives in seaweeds, the enzyme abietadienol/abietadienal oxidase is a multifunctional and multisubstrate cytochrome P450 enzyme [113], and could be involved in the biosynthesis of diverse diterpenes in *L. dendroidea* through oxidation steps.

The gibberellins are important endogenous growth regulators, well recognized in vascular plants. Diverse studies point to a gibberellin-like activity in extracts of seaweeds, which led to the broad utilization of seaweeds in the formulation of commercial plant fertilizers [114]. Recently, a chemical analysis of the extract of the brown seaweed *Ecklonia maxima* was able to reliably demonstrate for the first time the presence of gibberellins in seaweed [115]. The expression of candidate genes for gibberellin 20-oxidase and gibberellin 2-oxidase in *L. dendroidea* is the first evidence for the occurrence of gibberellins in a red seaweed, although a more detailed chemical evaluation is necessary. A comparison between the conserved domain hits found for some candidate genes from *L*. *dendroidea* and the sequences coding for gibberellin 20-oxidase and gibberellin 2-oxidase from *Arabidopsis thaliana* showed the presence of typical gibberellin synthase domains (available in the Supplementary Information).

## 4. Experimental Section

*Laurenciadendroidea* was sampled in the intertidal zone at Castelhanos beach in Anchieta municipality, Espírito Santo State, Brazil (20°51’40”S, 40°37’00”W) in 2008 and has been maintained in the laboratory since then. In order to establish a unialgal culture of this seaweed, the apices were successively excised and grown in sterile seawater enriched with 25% Provasoli solution [116]. These algal clones were treated with an antibiotic mix to reduce the bacteria in the culture (100 µg/mL ampicillin, 120 µg/mL streptomycin, and 60 µg/mL gentamicin) and were grown in sterile seawater with germanium dioxide (1 mg/L) and 50% Provasoli solution for 2 days before the experiment. The culture conditions were salinity 32 ± 1, temperature 22 ± 1 °C, irradiance 80 ± 5 µmol photons m^−2^·s^−1^, and 14 h light/10 h dark. To increase the scope of this work and maximize the detection of genes related to the biosynthesis of terpenes, we analyzed the trascriptome of *L*. *dendroidea* after exposure to dark and light conditions. Thus, one clone of this seaweed was sampled at the end of the dark period and the other was sampled after 10 h in the light.

The algal clones were separately ground in liquid nitrogen using a mortar and pestle to obtain a fine powder. The total RNA was extracted using the TRIzol reagent (Life Technologies-Invitrogen, Carlsbad, CA, USA) protocol. The DNA residues were eliminated with DNAse (RNAse free, PROMEGA, Madison, WI, USA), and the double-stranded cDNAs (ds cDNAs) were synthesized and amplified using the SMARTer cDNA synthesis kit and the Advantage2 polymerase (Clontech, Foster City, CA, USA) starting from 1 μg of total RNA. The optimal number of amplification cycles was determined to be 23. The PCR amplification products were purified using the NucleoSpin Extract II kit (Macherey-Nagel, Düren, Alemanha) and the ds cDNAs were eluted in nuclease-free water.

The ds cDNA libraries were prepared using the Nextera XT Sample Preparation Kit (Illumina, San Diego, CA, USA) and the size distribution was accessed using the 2100 Bioanalyzer (Agilent, Santa Clara, CA, USA) and the High Sensitivity DNA Kit (Agilent, Santa Clara, CA, USA). The accurate quantification of the libraries was accomplished using the 7500 Real Time PCR (Applied Biosystems, Foster City, CA, USA) and the KAPA Library Quantification Kit (Kapa Biosystems, Wilmington, MA, USA). Paired-end sequencing (2 × 150 bp and 2 × 250 bp) was performed on a MiSeq (Illumina, San Diego, CA, USA).

The sequences from each sample were preprocessed using the software Prinseq [117] to trim poly-A/T tails at least 20 bp long, to remove reads shorter than 35 bp, and to trim sequences with a quality score lower than 30. Then the sequences were assembled using the software Trinity, which is based on de Brujin graphs [118], and both contigs and singlets were used in the downstream analysis. To identify the transcripts associated to the synthesis of terpenoid compounds, we prospected the transcriptome of *L*. *dendroidea* using hidden markov models generated from the alignment of sequences available in the KEGG database through the HMMER 3.0 software [119]. Other searches using specific HMM profiles were based on the alignment of all vanadium-dependent bromoperoxidase sequences deposited in the protein database of NCBI and on the universal metal-binding domain (PF03936) and *N*-terminal domain (PF01397) of terpene synthases obtained from PFAM as previously described [120]. The sequences matching all these profiles were annotated through BLAST search against the NCBI-nr, PlantCyc, and Uniprot databases. The functional identifications were manually confirmed.

To perform the cytochemical labeling of the activity of HMGS and/or HMGR, the release of spores was induced in the laboratory by subjecting *L*. *dendroidea* sporophytes to light deprivation. The spores were maintained for 12 h in sterile seawater enriched with 50% Von Stosch solution [121] at 20 °C, and 60 μmol phtons m^2^·s^−1^. The viable spores that adhered to the bottom of the Petri dishes were selected for the experiment. Spores are proliferative and developing cells, which can better reveal the primary regions involved in mevalonic acid production.

The cytochemical labeling method, previously described by Curry (1987) [122], is based on the reaction catalyzed by the enzyme HMGS that converts acetyl CoA and acetoacetyl CoA in 3-hydroxy-3-methylglutaryl-CoA, which is subsequently reduced by HMGR. Both reactions produce free Coenzyme A-SH (CoA-SH) [123], which reacts with potassium ferricyanide, reducing it to ferrocyanide. The ferrocyanide then reacts with added uranyl acetate to form uranyl ferrocyanide, which precipitates and appears as a highly electron-dense material in transmission electron microscopy (TEM). In this way, the localization of HGMR is indistinguishable from that of HGMS because both reactions produce CoA-SH, which precipitates as uranyl ferrocyanide.

The spores of *L*. *dendroidea* were fixed for 30 min in 4% formaldehyde and 1% glutaraldehyde in 0.05 M sodium cacodylate buffer (pH 7.6) diluted in sterile seawater, followed by a buffer rinse (0.05 M cacodylate, pH 7.6). Subsequently, the algal spores were pre-incubated at room temperature for 20 min in 3 mM potassium ferrocyanide in 0.05 M cacodylate buffer (pH 7.6), followed by a buffer rinse [122]. Then, a group of spores was maintained for 45 min at room temperature with the complete incubation solution composed by acetyl-CoA sodium salt (0.8 mg·mL^−1^), acetoacetyl-CoA sodium salt (1.6 mg·mL^−1^), potassium ferricyanide (2.0 mg·mL^−1^), uranyl acetate (1.0 mg·mL^−1^), and sodium cacodylate buffer (0.05 M, pH 7.0); a second group received the same compounds except for the acetyl-CoA sodium salt (substrate control), and a third group received the same compounds that were in the complete incubation solution except for acetoacetyl-CoA sodium salt (reaction product control). The last two groups were used as a control to test for unspecific precipitations of uranyl ferrocyanide caused by the action of any enzyme other than HMGS and HMGR. After that, the algal spores were post-fixed for one hour at room temperature in 2% (w/v) osmium tetroxide in 0.05 M sodium cacodylate buffer (pH 7.0), followed by a buffer rinse. The algal spores were dehydrated in a crescent acetone series (up to 100%) and embedded in Spurr resin. The polymerization process was performed at 70 °C and the ultrathin sections (70 nm) were obtained in a Reichert ultramicrotome and collected on copper grids (300 mesh). The grids were contrasted with uranyl acetate and lead citrate and 50 spore cells from each treatment were observed in a JEOL 1200 EX transmission electron microscope (JEOL, Peabody, MA, USA).

## 5. Conclusions

*Laurencia dendroidea* expresses a suite of genes encoding terpene synthases that catalyze the chemical modifications of precursors resulting in the high diversity of terpenoid compounds known for this species. The unveiling of genes associated to the chemical defense against natural enemies and the cytochemical evidence for the occurrence of the mevalonate pathway in *L*. *dendroidea* provided a better understanding of the molecular and biochemical processes and the cell structures involved in the biosynthesis of these secondary compounds that affect the biological interactions of this seaweed in the marine environment. The present work offered valuable information toward future sustainable production of biotechnologically relevant terpenes from *L*. *dendroidea* using genetically modified organisms in fermentation processes.

## Supplementary Files

Supplementary File 1
