# Supplementary material for: New Insights on the Terpenome of the Red Seaweed Laurencia dendroidea (Florideophyceae, Rhodophyta)
Source: Mar Drugs. 2015 Feb 10;13(2):879–902. doi: 10.3390/md13020879 (PMC4344607; doi:10.3390/md13020879)
Supplement: Supplementary File 1 [file marinedrugs-13-00879-s001.pdf]

# Supplementary Information

The domain composition of sequences from *Laurencia dendroidea* that were annotated as terpene synthases and presented in Table 2 of the main manuscript were obtained through search for conserved domains using the NCBI Conserved Domain Database (CDD, [1]) and compared with the domain composition of corresponding sequences available in the SwissProt/UniProt and PlantCycDB databases. Analyzing the tables and figures below, we reinforce the Blast annotations presented in Table 2 of the main manuscript text, although biochemical and gene cloning approaches are necessary to prove these *in silico* identifications. For two of the 21 terpene synthase sequences from *Laurencia* (identified as nerolidol synthase and (+)-delta-cadinene synthase), we were not able to identify conserved domains, possibly because they were partial sequences. In the case of alpha-bisabolene synthase, we provide the domain composition of a reference sequence and the alignment of the sequence from *L. dendroidea* with this reference, showing a high similarity between them.

**Table S1.** List of domain hits for a putative (3*R*)-linalool synthase gene from *L. dendroidea*.

| Name | Accession | Description | Interval | E-Value |
|---|---|---|---|---|
| rad1 | TIGR00596 | DNA repair protein (rad1); All proteins in this family for which functions are known are ... | 1–51 | 1.04e-15 |

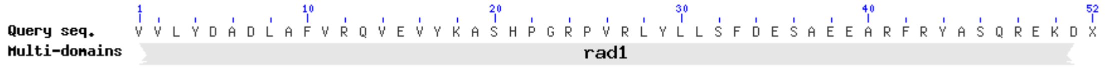


**Figure S1.** Conserved domains detected in a putative (3*R*)-linalool synthase gene from *L. dendroidea* (edited from the NCBI CDD database, [1]).

**Table S2.** List of domain hits for the (3R)-linalool synthase gene from *Zea mays mays* (GDQC-116173-MONOMER—PlantCycDB).

| Name | Accession | Description | Interval | E-Value |
|---|---|---|---|---|
| Terpene_cyclase_plant_C1 | cd00684 | Plant Terpene Cyclases, Class 1; This CD includes a diverse group of monomeric plant terpene ... | 1377–1833 | 9.57e-107 |
| ERCC4 | pfam02732 | ERCC4 domain; This domain is a family of nucleases. The family includes EME1 which is an ... | 732–859 | 2.10e-28 |
| rad1 | TIGR00596 | DNA repair protein (rad1); All proteins in this family for which functions are known are ... | 88–933 | 0e+00 |

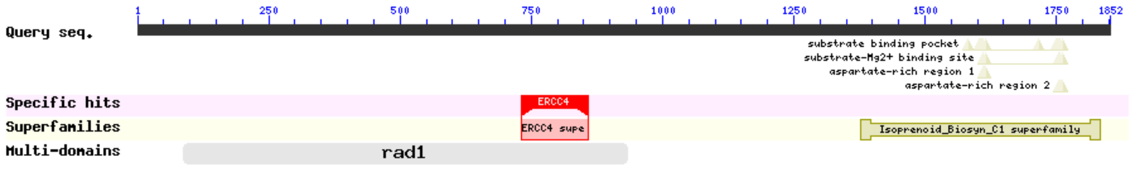


**Figure S2.** Conserved domains detected in the (3R)-linalool synthase gene from *Zea mays mays* (GDQC-116173-MONOMER—PlantCycDB) (edited from the NCBI CDD database, [1]).

**Table S3.** List of domain hits for a putative (+)-trans-carveol dehydrogenase gene from *L. dendroidea*.

| Name | Accession | Description | Interval | E-value |
|---|---|---|---|---|
| CR_SDR_c | cd08936 | Porcine peroxisomal carbonyl reductase like, classical (c) SDR; This subgroup contains porcine ... | 17–272 | 1.05e-91 |
| PRK05875 | PRK05875 | short chain dehydrogenase; Provisional | 11–267 | 2.12e-39 |
| adh_short_C2 | pfam13561 | Enoyl-(Acyl carrier protein) reductase; | 22–268 | 1.19e-35 |
| PLN02730 | PLN02730 | enoyl-[acyl-carrier-protein] reductase | 85–262 | 3.56e-14 |
| sepiapter_red | TIGR01500 | sepiapterin reductase; This model describes sepiapterin reductase, a member of the short chain ... | 17–201 | 5.40e-06 |
| PKS_KR | smart00822 | This enzymatic domain is part of bacterial polyketide synthases; It catalyses the first step ... | 19–167 | 2.88e-04 |
| fabG | PRK07231 | 3-ketoacyl-(acyl-carrier-protein) reductase; Provisional | 17–267 | 2.43e-77 |
| FabG | COG1028 | Dehydrogenases with different specificities (related to short-chain alcohol dehydrogenases) ... | 14–267 | 4.59e-61 |
| 3oxo_ACP_reduc | TIGR01830 | 3-oxoacyl-(acyl-carrier-protein) reductase; This model represents 3-oxoacyl-[ACP] reductase, ... | 18–267 | 3.80e-55 |
| adh_short | pfam00106 | short chain dehydrogenase; This family contains a wide variety of dehydrogenases. | 17–185 | 4.24e-25 |
| PLN02253 | PLN02253 | xanthoxin dehydrogenase | 10–267 | 3.65e-18 |
| Se_ygfK | TIGR03315 | putative selenate reductase, YgfK subunit; Members of this protein family are YgfK, predicted ... | 6–63 | 5.85e-03 |

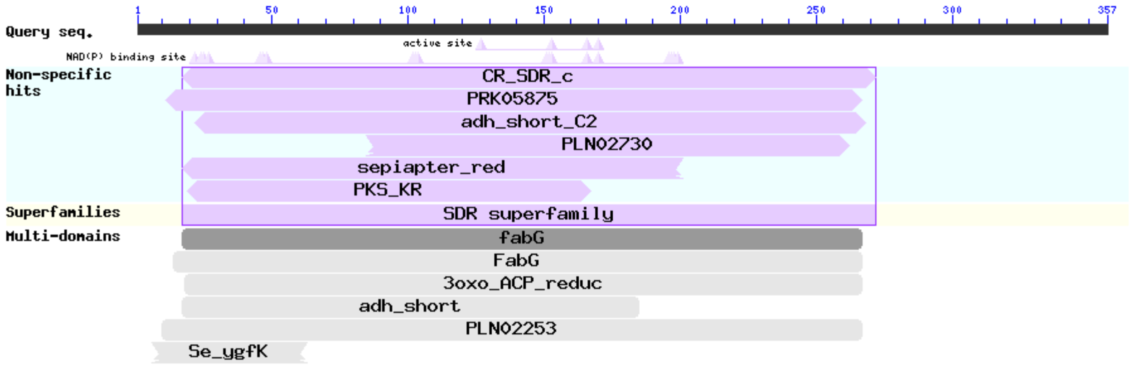


**Figure S3.** Conserved domains detected in a putative (+)-trans-carveol dehydrogenase gene from *L. dendroidea* (edited from the NCBI CDD database, [1]).

**Table S4.** List of domain hits for the (+)-trans-carveol dehydrogenase gene from *Mentha piperita* (sp|Q5C9I9|ISPD_MENPI).

| Name | Accession | Description | Interval | E-Value |
|---|---|---|---|---|
| secoisolariciresinol-DH_like_SDR_c | cd05326 | secoisolariciresinol dehydrogenase (secoisolariciresinol-DH)-like, classical (c) SDRs; | 6–255 | 4.43e-70 |
| PRK06947 | PRK06947 | glucose-1-dehydrogenase; Provisional | 10–254 | 1.34e-28 |
| adh_short_C2 | pfam13561 | Enoyl-(Acyl carrier protein) reductase; | 16–255 | 1.75e-19 |
| PLN00015 | PLN00015 | protochlorophyllide reductase | 13–102 | 3.87e-10 |
| PKS_KR | smart00822 | This enzymatic domain is part of bacterial polyketide synthases; It catalyses the first step ... | 10–124 | 5.58e-09 |
| LPOR | TIGR01289 | light-dependent protochlorophyllide reductase; This model represents the light-dependent, | 12–101 | 3.14e-06 |
| PLN02253 | PLN02253 | xanthoxin dehydrogenase | 3–257 | 2.86e-63 |
| PRK08324 | PRK08324 | short chain dehydrogenase; Validated | 1–255 | 4.79e-61 |
| FabG | COG1028 | Dehydrogenases with different specificities (related to short-chain alcohol dehydrogenases) ... | 6–255 | 1.64e-50 |
| RhaD_aldol-ADH | TIGR02632 | rhamnulose-1-phosphate aldolase/alcohol dehydrogenase; | 1–255 | 3.70e-45 |
| adh_short | pfam00106 | short chain dehydrogenase; This family contains a wide variety of dehydrogenases. | 10–178 | 4.18e-20 |

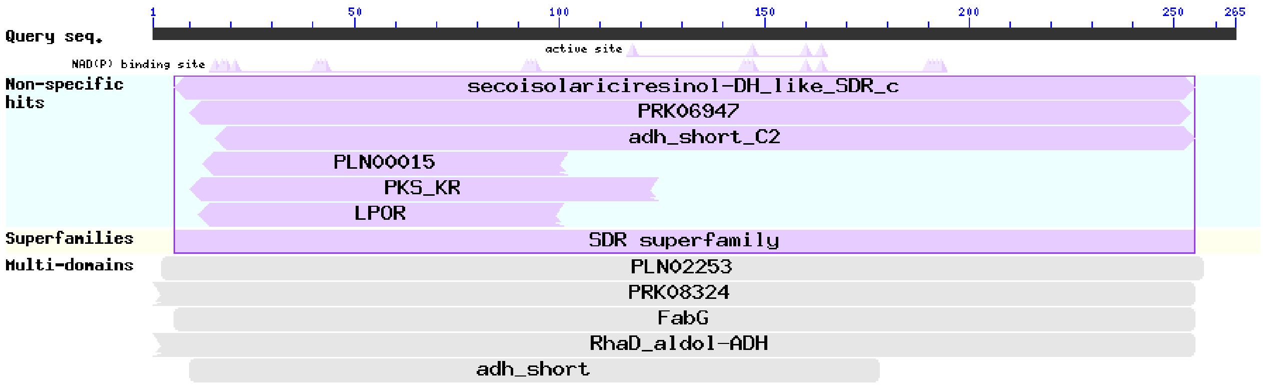


**Figure S4.** Conserved domains detected in the (+)-trans-carveol dehydrogenase gene from *Mentha piperita* (sp|Q5C9I9|ISPD_MENPI) (edited from the NCBI CDD database, [1]).

**Table S5.** List of domain hits for a putative (+)-pulegone reductase gene from *L. dendroidea*.

| Name | Accession | Description | Interval | E-Value |
|---|---|---|---|---|
| PGDH | cd05288 | Prostaglandin dehydrogenases; Prostaglandins and related eicosanoids are metabolized by the ... | 1–293 | 9.21e-128 |
| leukotriene_B4_DH_like | cd08294 | 13-PGR is a bifunctional enzyme with delta-13 15-prostaglandin reductase and leukotriene B4 12 ... | 14–294 | 1.49e-74 |
| double_bond_reductase_like | cd08295 | Arabidopsis alkenal double bond reductase and leukotriene B4 12-hydroxydehydrogenase; This ... | 1–295 | 7.02e-73 |
| PTGR2 | cd08293 | Prostaglandin reductase; Prostaglandins and related eicosanoids are metabolized by the ... | 1–294 | 3.04e-65 |
| Mgc45594_like | cd08250 | Mgc45594 gene product and other MDR family members; Includes Human Mgc45594 gene product of ... | 28–294 | 1.18e-48 |
| QOR1 | cd08241 | Quinone oxidoreductase (QOR); QOR catalyzes the conversion of a quinone + NAD(P)H to a ... | 29–294 | 1.89e-39 |
| p53_inducible_oxidoreductase | cd05276 | PIG3 p53-inducible quinone oxidoreductase; PIG3 p53-inducible quinone oxidoreductase, a medium ... | 53–293 | 9.65e-29 |
| zeta_crystallin | cd08253 | Zeta-crystallin with NADP-dependent quinone reductase activity (QOR); Zeta-crystallin is a eye ... | 65–295 | 6.42e-27 |
| MDR_like_2 | cd05289 | alcohol dehydrogenase and quinone reductase-like medium chain degydrogenases/reductases; ... | 45–293 | 5.28e-26 |
| Zn_ADH_like1 | cd08266 | Alcohol dehydrogenases of the MDR family; This group contains proteins related to the ... | 85–295 | 9.64e-24 |

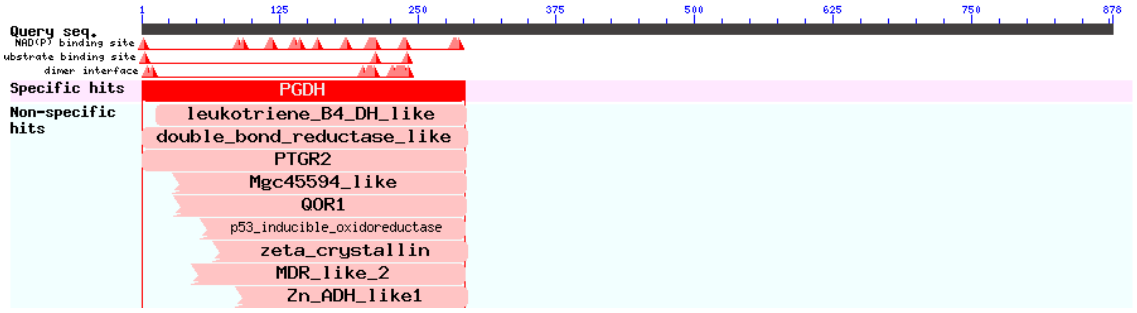


**Figure S5.** Conserved domains detected in a putative (+)-pulegone reductase gene from *L. dendroidea* (edited from the NCBI CDD database [1]).

**Table S6.** List of domain hits for the (+)-pulegone reductase gene from *Mentha piperita* (sp|Q6WAU0|PULR_MENPI).

| Name | Accession | Description | Interval | E-Value |
|---|---|---|---|---|
| double_bond_reductase_like | cd08295 | Arabidopsis alkenal double bond reductase and leukotriene B4 12-hydroxydehydrogenase; This ... | 2–339 | 0e+00 |
| PGDH | cd05288 | Prostaglandin dehydrogenases; Prostaglandins and related eicosanoids are metabolized by the ... | 3–337 | 1.06e-144 |
| leukotriene_B4_DH_like | cd08294 | 13-PGR is a bifunctional enzyme with delta-13 15-prostaglandin reductase and leukotriene B4 12 ... | 2–339 | 1.23e-104 |
| PTGR2 | cd08293 | Prostaglandin reductase; Prostaglandins and related eicosanoids are metabolized by the ... | 3–339 | 3.91e-65 |
| Mgc45594_like | cd08250 | Mgc45594 gene product and other MDR family members; Includes Human Mgc45594 gene product of ... | 137–337 | 7.60e-47 |
| zeta_crystallin | cd08253 | Zeta-crystallin with NADP-dependent quinone reductase activity (QOR); Zeta-crystallin is a eye ... | 29–283 | 3.04e-28 |
| MDR | cd05188 | Medium chain reductase/dehydrogenase (MDR)/zinc-dependent alcohol dehydrogenase-like family; ... | 94–297 | 9.69e-27 |
| p53_inducible_oxidoreductase | cd05276 | PIG3 p53-inducible quinone oxidoreductase; PIG3 p53-inducible quinone oxidoreductase, a medium ... | 138–307 | 1.44e-25 |
| MDR_like_2 | cd05289 | alcohol dehydrogenase and quinone reductase-like medium chain degydrogenases/reductases; ... | 31–323 | 3.42e-25 |
| ADH_zinc_N | pfam00107 | Zinc-binding dehydrogenase; | 16–298 | 3.91e-21 |

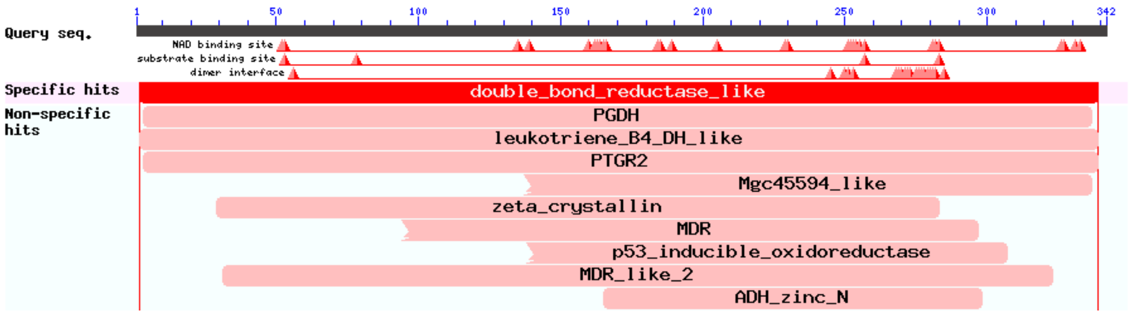


**Figure S6.** Conserved domains detected in the (+)-pulegone reductase gene from *Mentha piperita* (sp|Q6WAU0|PULR_MENPI) (edited from the NCBI CDD database, [1]).

**Table S7.** List of domain hits for a putative (−)-isopiperitenone reductase gene from *L. dendroidea*.

| Name | Accession | Description | Interval | E-Value |
|---|---|---|---|---|
| 17beta-HSD1_like_SDR_c | cd05356 | 17-beta-hydroxysteroid dehydrogenases (17beta-HSDs) types -1, -3, and -12, -like, classical (c) ... | 34–146 | 3.56e-31 |
| PRK08339 | PRK08339 | short chain dehydrogenase; Provisional | 27–112 | 1.20e-09 |
| sepiapter_red | TIGR01500 | sepiapterin reductase; This model describes sepiapterin reductase, a member of the short chain ... | 37–119 | 3.85e-06 |
| DltE | COG0300 | Short-chain dehydrogenases of various substrate specificities [General function prediction ... | 32–146 | 7.97e-29 |
| PLN02780 | PLN02780 | ketoreductase/oxidoreductase | 14–146 | 7.50e-23 |
| fabG | PRK07666 | 3-ketoacyl-(acyl-carrier-protein) reductase; Provisional | 37–145 | 3.61e-17 |
| 23BDH | TIGR02415 | acetoin reductases; One member of this family, as characterized in Klebsiella terrigena, is ... | 37–145 | 3.62e-12 |
| adh_short | pfam00106 | short chain dehydrogenase; This family contains a wide variety of dehydrogenases. | 36–145 | 1.50e-09 |

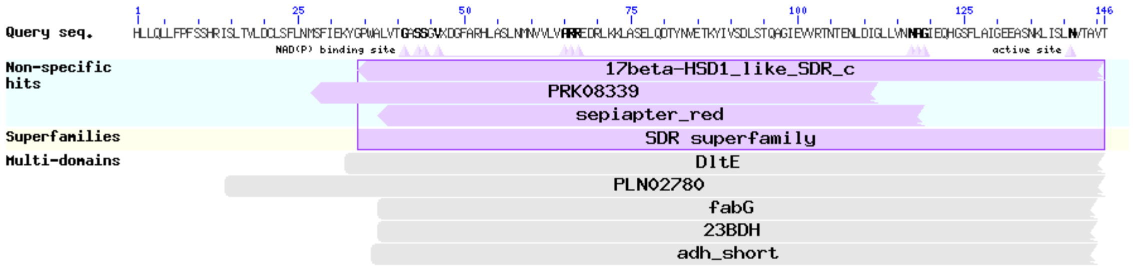


**Figure S7.** Conserved domains detected in a putative (−)-isopiperitenone reductase gene from *L. dendroidea* (edited from the NCBI CDD database, [1]).

**Table S8.** List of domain hits for the (−)-isopiperitenone reductase gene from *Mentha piperita* (sp|Q6WAU1|IPIPR_MENPI).

| Name | Accession | Description | Interval | E-Value |
|---|---|---|---|---|
| carb_red_PTCR-like_SDR_c | cd05324 | Porcine testicular carbonyl reductase (PTCR)-like, classical (c) SDRs; PTCR is a classical SDR ... | 6–311 | 1.99e-74 |
| PRK05854 | PRK05854 | short chain dehydrogenase; Provisional | 6–96 | 4.90e-15 |
| adh_short_C2 | pfam13561 | Enoyl-(Acyl carrier protein) reductase; | 15–126 | 4.28e-11 |
| PKS_KR | smart00822 | This enzymatic domain is part of bacterial polyketide synthases; It catalyses the first step ... | 9–96 | 8.02e-11 |
| sepiapter_red | TIGR01500 | sepiapterin reductase; This model describes sepiapterin reductase, a member of the short chain ... | 7–120 | 1.78e-06 |

**Table S8.** *Cont.*

| | | | | |
|---|---|---|---|---|
| PLN00015 | PLN00015 | protochlorophyllide reductase | 9–96 | 1.60e-03 |
| PRK12939 | PRK12939 | short chain dehydrogenase; Provisional | 6–299 | 2.99e-15 |
| FabG | COG1028 | Dehydrogenases with different specificities (related to short-chain alcohol dehydrogenases) . | 8–300 | 4.86e-27 |
| adh_short | pfam00106 | short chain dehydrogenase; This family contains a wide variety of dehydrogenases. | 8–138 | 3.61e-19 |

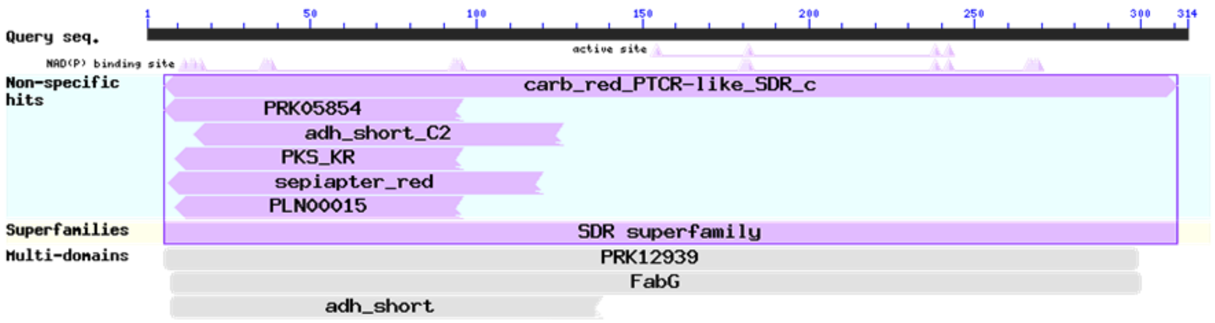


**Figure S8.** Conserved domains detected in the (−)-isopiperitenone reductase gene from *Mentha piperita* (sp|Q6WAU1|IPIPR_MENPI) (edited from the NCBI CDD database, [1]).

**Table S9.** List of domain hits for a putative secologanin synthase gene from *L. dendroidea*.

| Name | Accession | Description | Interval | E-Value |
|---|---|---|---|---|
| p450 | pfam00067 | Cytochrome P450; Cytochrome P450s are haem-thiolate proteins involved in the oxidative | 160–384 | 2.32e-29 |
| CypX | COG2124 | Cytochrome P450 [Secondary metabolites biosynthesis, transport, and catabolism] | 13–378 | 2.81e-25 |
| PLN02290 | PLN02290 | cytokinin trans-hydroxylase | 170–351 | 8.00e-17 |
| PLN02169 | PLN02169 | fatty acid (omega-1)-hydroxylase/midchain alkane hydroxylase | 168–356 | 8.21e-17 |
| PLN03195 | PLN03195 | fatty acid omega-hydroxylase; Provisional | 167–378 | 9.71e-15 |
| PLN02426 | PLN02426 | cytochrome P450, family 94, subfamily C protein | 168–370 | 6.50e-14 |
| PLN00168 | PLN00168 | Cytochrome P450; Provisional | 160–356 | 7.30e-14 |
| PLN02936 | PLN02936 | epsilon-ring hydroxylase | 162–380 | 1.74e-13 |
| PLN02687 | PLN02687 | flavonoid 3′-monooxygenase | 159–344 | 6.33e-09 |
| PLN02655 | PLN02655 | ent-kaurene oxidase | 160–378 | 1.04e-07 |
| PLN00110 | PLN00110 | flavonoid 3′,5′-hydroxylase (F3′5′H); Provisional | 154–300 | 6.62e-07 |
| PTZ00404 | PTZ00404 | cytochrome P450; Provisional | 164–359 | 7.19e-07 |
| PLN02183 | PLN02183 | ferulate 5-hydroxylase | 160–298 | 1.08e-06 |
| PLN03018 | PLN03018 | homomethionine *N*-hydroxylase | 155–349 | 1.21e-06 |
| PLN03112 | PLN03112 | cytochrome P450 family protein; Provisional | 172–300 | 3.83e-06 |
| PLN02971 | PLN02971 | tryptophan *N*-hydroxylase | 157–357 | 5.06e-06 |
| PLN02966 | PLN02966 | cytochrome P450 83A1 | 157–338 | 5.44e-06 |
| PLN03234 | PLN03234 | cytochrome P450 83B1; Provisional | 144–345 | 1.74e-05 |
| PLN02394 | PLN02394 | trans-cinnamate 4-monooxygenase | 178–380 | 2.89e-04 |
| PLN02302 | PLN02302 | ent-kaurenoic acid oxidase | 152–359 | 4.88e-04 |
| PLN02774 | PLN02774 | brassinosteroid-6-oxidase | 159–361 | 6.03e-03 |

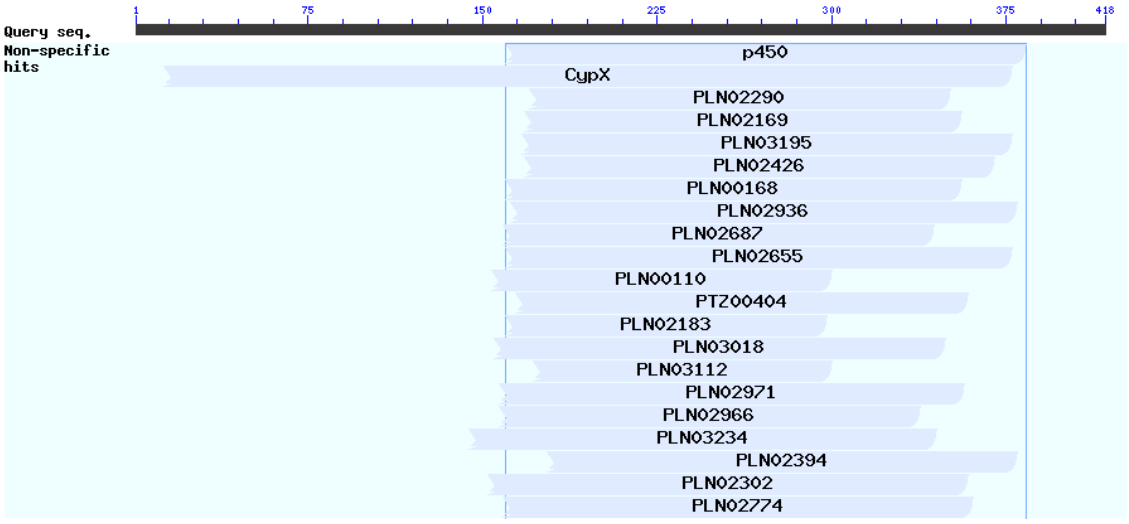


**Figure S9.** Conserved domains detected in a putative secologanin synthase gene from *L. dendroidea* (edited from the NCBI CDD database, [1]).

**Table S10.** List of domain hits for the secologanin synthase gene from *Catharanthus roseus* (sp|Q05047|C72A1_CATRO).

| Name | Accession | Description | Interval | E-Value |
|---|---|---|---|---|
| PLN02290 | PLN02290 | cytokinin trans-hydroxylase | 5–524 | 2.05e-129 |
| p450 | pfam00067 | Cytochrome P450; Cytochrome P450s are haem-thiolate proteins involved in the oxidative ... | 102–497 | 4.56e-82 |
| CypX | COG2124 | Cytochrome P450 [Secondary metabolites biosynthesis, transport, and catabolism] | 114–521 | 3.87e-50 |
| PLN02936 | PLN02936 | epsilon-ring hydroxylase | 100–497 | 3.71e-41 |
| PLN02687 | PLN02687 | flavonoid 3′-monooxygenase | 190–496 | 2.09e-26 |
| PLN02426 | PLN02426 | cytochrome P450, family 94, subfamily C protein | 260–503 | 2.27e-26 |
| PTZ00404 | PTZ00404 | cytochrome P450; Provisional | 64–493 | 7.42e-24 |
| PLN03195 | PLN03195 | fatty acid omega-hydroxylase; Provisional | 110–497 | 8.36e-24 |
| PLN02302 | PLN02302 | ent-kaurenoic acid oxidase | 88–507 | 3.89e-23 |
| PLN02183 | PLN02183 | ferulate 5-hydroxylase | 208–495 | 8.15e-23 |
| PLN03112 | PLN03112 | cytochrome P450 family protein; Provisional | 93–497 | 1.07e-21 |
| PLN02169 | PLN02169 | fatty acid (omega-1)-hydroxylase/midchain alkane hydroxylase | 260–521 | 1.98e-21 |
| PLN02655 | PLN02655 | ent-kaurene oxidase | 228–491 | 2.30e-21 |
| PLN02966 | PLN02966 | cytochrome P450 83A1 | 183–495 | 8.06e-19 |
| PLN03234 | PLN03234 | cytochrome P450 83B1; Provisional | 164–495 | 2.81e-18 |
| PLN00168 | PLN00168 | Cytochrome P450; Provisional | 314–497 | 3.95e-16 |
| PLN02987 | PLN02987 | Cytochrome P450, family 90, subfamily A | 223–493 | 2.87e-15 |
| PLN02971 | PLN02971 | tryptophan *N*-hydroxylase | 242–498 | 3.03e-15 |
| PLN02394 | PLN02394 | trans-cinnamate 4-monooxygenase | 332–491 | 1.33e-14 |
| PLN00110 | PLN00110 | flavonoid 3′,5′-hydroxylase (F3′5′H); Provisional | 247–491 | 6.65e-12 |
| PLN02500 | PLN02500 | cytochrome P450 90B1 | 272–496 | 1.01e-11 |
| PLN03018 | PLN03018 | homomethionine *N*-hydroxylase | 150–522 | 3.32e-11 |
| PLN02774 | PLN02774 | brassinosteroid-6-oxidase | 107–494 | 4.36e-11 |

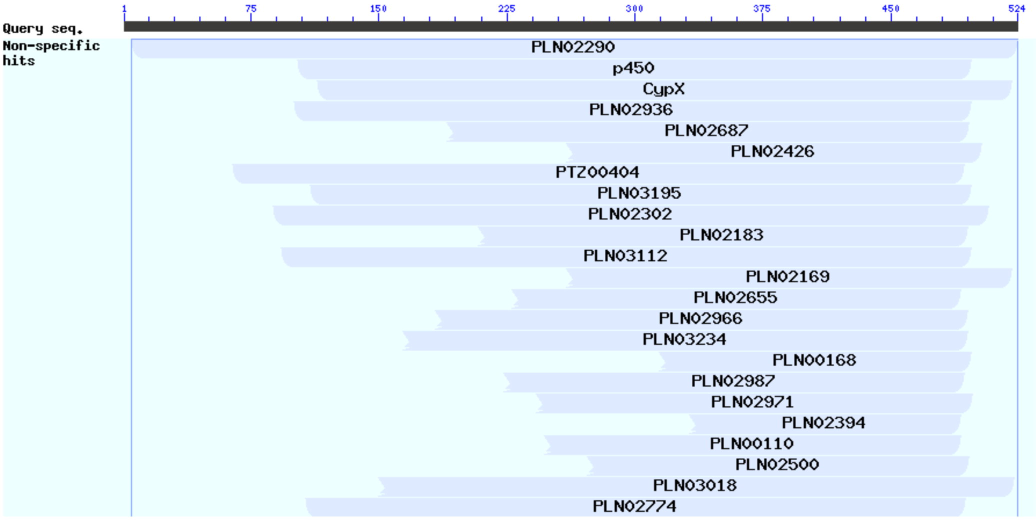


**Figure S10.** Conserved domains detected in the secologanin synthase gene from *Catharanthus roseus* (sp|Q05047|C72A1_CATRO) (edited from the NCBI CDD database, [1]).

**Table S11.** List of domain hits for a putative farnesyl-diphosphate farnesyltransferase gene from *L. dendroidea*.

| Name | Accession | Description | Interval | E-Value |
|---|---|---|---|---|
| squal_synth | TIGR01559 | farnesyl-diphosphate farnesyltransferase; This model describes farnesyl-diphosphate ... | 139–411 | 1.00e-104 |
| Trans_IPPS_HH | cd00683 | Trans-Isoprenyl Diphosphate Synthases, head-to-head; These trans-Isoprenyl Diphosphate ... | 165–364 | 2.49e-47 |
| SQS_PSY | pfam00494 | Squalene/phytoene synthase; | 158–361 | 1.01e-37 |
| ERG9 | COG1562 | Phytoene/squalene synthetase [Lipid metabolism] | 180–370 | 2.86e-35 |
| Trans_IPPS | cd00867 | Trans-Isoprenyl Diphosphate Synthases; Trans-Isoprenyl Diphosphate Synthases (Trans_IPPS) of ... | 179–286 | 1.13e-17 |
| Isoprenoid_Biosyn_C1 | cd00385 | Isoprenoid Biosynthesis enzymes, Class 1; Superfamily of trans-isoprenyl diphosphate synthases ... | 174–325 | 4.43e-17 |
| HpnD | TIGR03465 | squalene synthase HpnD; The genes of this family are often found in the same genetic locus ... | 193–275 | 1.41e-08 |
| PLN02632 | PLN02632 | phytoene synthase | 185–275 | 4.93e-07 |

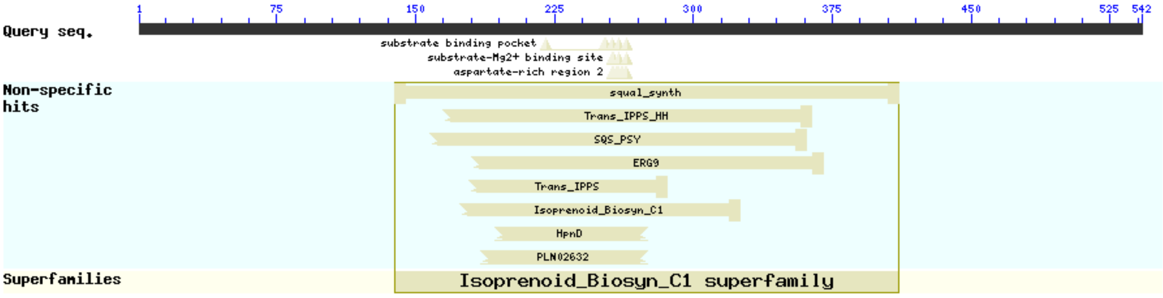


**Figure S11.** Conserved domains detected in a putative farnesyl-diphosphate farnesyltransferase gene from *L. dendroidea* (edited from the NCBI CDD database, [1]).

**Table S12.** List of domain hits for the farnesyl-diphosphate farnesyltransferase gene from *Scheffersomyces stipitis* (tr|A3LTQ5|A3LTQ5_PICST).

| Name | Accession | Description | Interval | E-value |
|---|---|---|---|---|
| Trans_IPPS_HH | cd00683 | Trans-Isoprenyl Diphosphate Synthases, head-to-head; These trans-Isoprenyl Diphosphate ... | 40–333 | 2.34e-74 |
| squal_synth | TIGR01559 | farnesyl-diphosphate farnesyltransferase; This model describes farnesyl-diphosphate ... | 39–381 | 0e+00 |
| ERG9 | COG1562 | Phytoene/squalene synthetase [Lipid metabolism] | 39–342 | 1.08e-57 |
| SQS_PSY | pfam00494 | Squalene/phytoene synthase; | 48–329 | 5.08e-48 |
| PLN02632 | PLN02632 | phytoene synthase | 170–242 | 9.10e-07 |

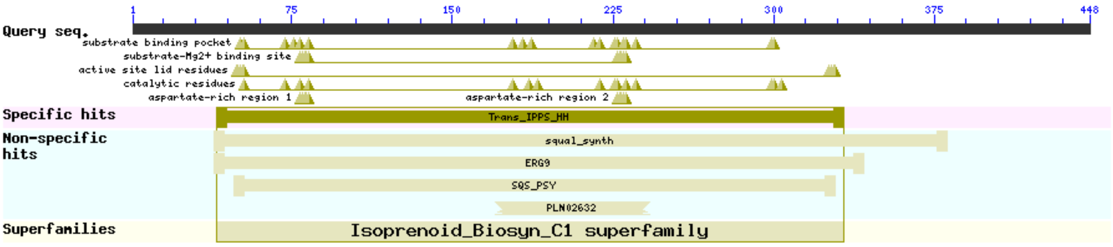


**Figure S12.** Conserved domains detected in the farnesyl-diphosphate farnesyltransferase gene from *Scheffersomyces stipitis* (tr|A3LTQ5|A3LTQ5_PICST) (edited from the NCBI CDD database, [1]).

**Table S13.** List of domain hits for a putative squalene monooxygenase gene from *L. dendroidea*.

| Name | Accession | Description | Interval | E-Value |
|---|---|---|---|---|
| SE | pfam08491 | Squalene epoxidase; This domain is found in squalene epoxidase (SE) and related proteins which ... | 11–258 | 2.16e-101 |
| PRK07608 | PRK07608 | ubiquinone biosynthesis hydroxylase family protein; Provisional | 100–200 | 3.03e-04 |
| COQ6 | TIGR01989 | ubiquinone biosynthesis monooxygenase COQ6; This model represents the monooxygenase ... | 110–198 | 7.59e-03 |
| PTZ00367 | PTZ00367 | squalene epoxidase; Provisional | 15–275 | 2.05e-68 |
| PLN02985 | PLN02985 | squalene monooxygenase | 30–275 | 6.46e-56 |
| UbiH | COG0654 | 2-polyprenyl-6-methoxyphenol hydroxylase and related FAD-dependent oxidoreductases [Coenzyme ... | 16–205 | 7.72e-12 |
| PRK07045 | PRK07045 | putative monooxygenase; Reviewed | 114–185 | 1.84e-04 |

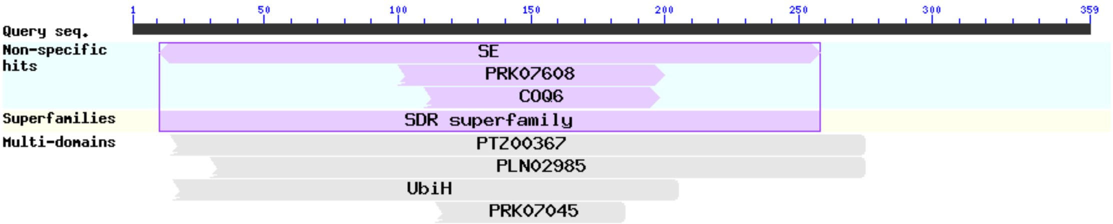


**Figure S13.** Conserved domains detected in a putative squalene monooxygenase gene from *L. dendroidea* (edited from the NCBI CDD database, [1]).

**Table S14.** List of domain hits for the squalene monooxygenase gene from *Arabidopsis thaliana* (sp|O65403|ERG13_ARATH).

| Name | Accession | Description | Interval | E-value |
|---|---|---|---|---|
| SE | pfam08491 | Squalene epoxidase; This domain is found in squalene epoxidase (SE) and related proteins which ... | 195–472 | 2.27e-124 |
| PRK08773 | PRK08773 | 2-octaprenyl-3-methyl-6-methoxy-1,4-benzoquinol hydroxylase; Validated | 40–364 | 3.79e-09 |
| NAD_binding_8 | pfam13450 | NAD(P)-binding Rossmann-like domain; | 48–74 | 5.95e-07 |
| PRK12834 | PRK12834 | putative FAD-binding dehydrogenase; Reviewed | 44–105 | 1.76e-06 |
| THI4 | COG1635 | Ribulose 1,5-bisphosphate synthetase, converts PRPP to RuBP, flavoprotein [Carbohydrate ... | 44–76 | 2.60e-05 |
| PLN02661 | PLN02661 | Putative thiazole synthesis | 44–74 | 3.21e-03 |
| PTZ00383 | PTZ00383 | malate:quinone oxidoreductase; Provisional | 25–74 | 5.01e-03 |
| PLN02985 | PLN02985 | squalene monooxygenase | 1–515 | 0e+00 |
| PTZ00367 | PTZ00367 | squalene epoxidase; Provisional | 6–499 | 3.11e-86 |
| UbiH | COG0654 | 2-polyprenyl-6-methoxyphenol hydroxylase and related FAD-dependent oxidoreductases [Coenzyme ... | 44–439 | 6.61e-36 |

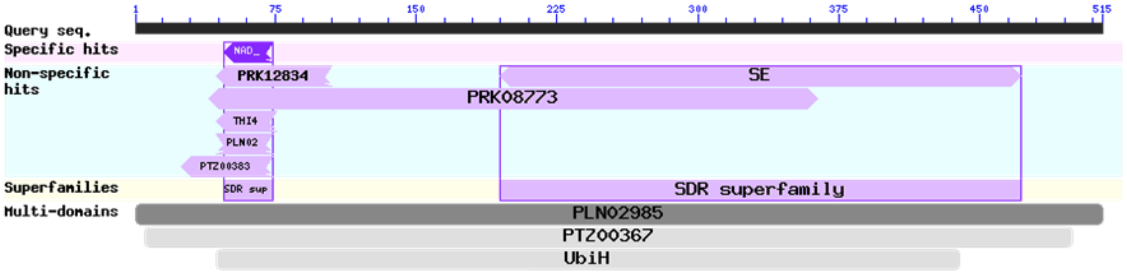


**Figure S14.** Conserved domains detected in the squalene monooxygenase gene from *Arabidopsis thaliana* (sp|O65403|ERG13_ARATH) (edited from the NCBI CDD database, [1]).

**Table S15.** List of domain hits for a putative squalene-hopene/tetraprenyl-beta-curcumene cyclase gene from *L. dendroidea*.

| Name | Accession | Description | Interval | E-value |
|---|---|---|---|---|
| SQCY_1 | cd02892 | Squalene cyclase (SQCY) domain subgroup 1; found in class II terpene cyclases that have an ... | 1–363 | 5.98e-143 |
| SQCY | cd02889 | Squalene cyclase (SQCY) domain; found in class II terpene cyclases that have an alpha 6— ... | 24–363 | 8.74e-126 |
| ISOPREN_C2_like | cd00688 | This group contains class II terpene cyclases, protein prenyltransferases beta subunit, two ... | 1–363 | 6.47e-39 |
| Prenyltrans | pfam00432 | Prenyltransferase and squalene oxidase repeat; | 207–240 | 3.26e-04 |
| SQCY_1 | cd02892 | Squalene cyclase (SQCY) domain subgroup 1; found in class II terpene cyclases that have an ... | 207–274 | 4.34e-03 |
| Prenyltrans_2 | pfam13249 | Prenyltransferase-like; | 211–331 | 7.97e-09 |
| Prenyltrans_2 | pfam13249 | Prenyltransferase-like; | 134–275 | 5.74e-08 |

**Table S15.** *Cont.*

| | | | | |
|---|---|---|---|---|
| squalene_cyclas | TIGR01787 | squalene/oxidosqualene cyclases; This family of enzymes catalyzes the cyclization of the ... | 3–363 | 1.19e-117 |
| PLN03012 | PLN03012 | Camelliol C synthase | 3–363 | 1.41e-106 |
| PLN02993 | PLN02993 | lupeol synthase | 3–369 | 4.48e-97 |
| osq_cycl | TIGR03463 | 2,3-oxidosqualene cyclase; This model identifies 2,3-oxidosqualene cyclases from Stigmatella ... | 1–361 | 1.37e-91 |
| SqhC | COG1657 | Squalene cyclase [Lipid metabolism] | 3–367 | 4.95e-56 |
| hopene_cyclase | TIGR01507 | squalene-hopene cyclase; SHC is an essential prokaryotic gene in hopanoid (triterpenoid) ... | 5–369 | 9.84e-30 |
| squa_tetra_cyc | TIGR04277 | squalene--tetrahymanol cyclase; This enzyme, also called squalene--tetrahymanol cyclase, ... | 126–363 | 2.76e-10 |
| Prenyltrans_1 | pfam13243 | Prenyltransferase-like; | 207–274 | 1.62e-08 |
| Prenyltrans_2 | pfam13249 | Prenyltransferase-like; | 83–224 | 1.02e-03 |
| Prenyltrans_1 | pfam13243 | Prenyltransferase-like; | 129–270 | 1.31e-03 |

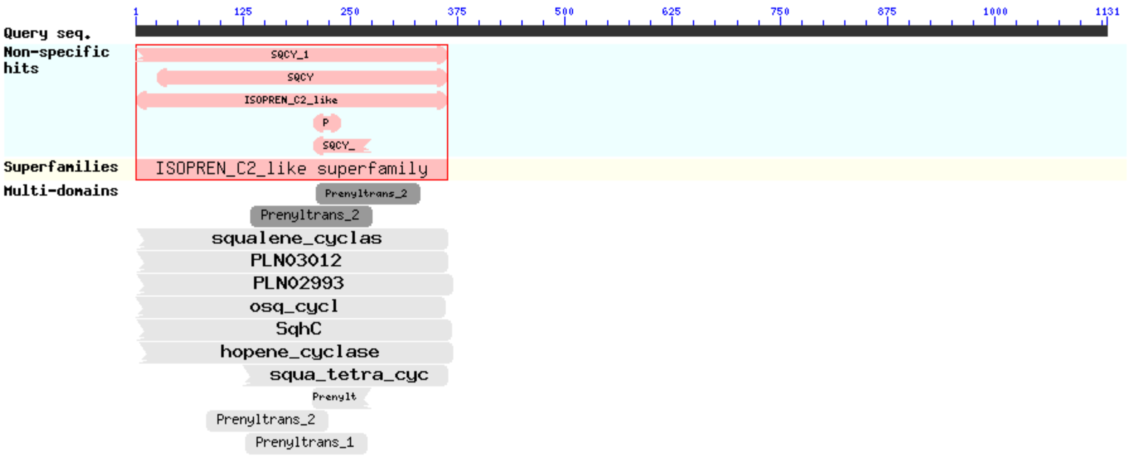


**Figure S15.** Conserved domains detected in a putative squalene-hopene/tetraprenyl-beta-curcumene cyclase gene from *L. dendroidea*, (edited from the NCBI CDD database, [1]).

**Table S16.** List of domain hits for the squalene-hopene/tetraprenyl-beta-curcumene cyclase gene from *Bradyrhizobium* sp. (tr|A4Z167 A4Z167_BRASO).

| **Name** | **Accession** | **Description** | **Interval** | **E-Value** |
|---|---|---|---|---|
| SQCY_1 | cd02892 | Squalene cyclase (SQCY) domain subgroup 1; found in class II terpene cyclases that have an ... | 26–677 | 0e+00 |
| SQCY | cd02889 | Squalene cyclase (SQCY) domain; found in class II terpene cyclases that have an alpha 6—... | 332–676 | 8.79e-130 |
| ISOPREN_C2_like | cd00688 | This group contains class II terpene cyclases, protein prenyltransferases beta subunit, two ... | 326–677 | 9.84e-39 |
| SQCY | cd02889 | Squalene cyclase (SQCY) domain; found in class II terpene cyclases that have an alpha 6— ... | 26–375 | 3.52e-08 |

**Table S16.** *Cont.*

| | | | | |
|---|---|---|---|---|
| ISOPREN_C2_like | cd00688 | This group contains class II terpene cyclases, protein prenyltransferases beta subunit, two ... | 26–410 | 7.38e-08 |
| SQCY | cd02889 | Squalene cyclase (SQCY) domain; found in class II terpene cyclases that have an alpha 6— ... | 18–97 | 1.79e-07 |
| SQCY_1 | cd02892 | Squalene cyclase (SQCY) domain subgroup 1; found in class II terpene cyclases that have an ... | 23–96 | 9.72e-07 |
| ISOPREN_C2_like | cd00688 | This group contains class II terpene cyclases, protein prenyltransferases beta subunit, two ... | 17–132 | 4.24e-05 |
| SQCY | cd02889 | Squalene cyclase (SQCY) domain; found in class II terpene cyclases that have an alpha 6— ... | 17–129 | 8.21e-05 |
| SQCY_1 | cd02892 | Squalene cyclase (SQCY) domain subgroup 1; found in class II terpene cyclases that have an ... | 17–129 | 1.16e-04 |
| Prenyltrans | pfam00432 | Prenyltransferase and squalene oxidase repeat; | 493–535 | 3.52e-04 |
| Prenyltrans | pfam00432 | Prenyltransferase and squalene oxidase repeat; | 541–583 | 5.36e-04 |
| hopene_cyclase | TIGR01507 | squalene-hopene cyclase; SHC is an essential prokaryotic gene in hopanoid (triterpenoid) ... | 26–676 | 0e+00 |
| squalene_cyclas | TIGR01787 | squalene/oxidosqualene cyclases; This family of enzymes catalyzes the cyclization of the ... | 25–676 | 1.99e-157 |
| SqhC | COG1657 | Squalene cyclase [Lipid metabolism] | 110–683 | 9.16e-74 |
| squa_tetra_cyc | TIGR04277 | squalene—tetrahymanol cyclase; This enzyme, also called squalene—tetrahymanol cyclase, ... | 77–681 | 6.52e-61 |
| osq_cycl | TIGR03463 | 2,3-oxidosqualene cyclase; This model identifies 2,3-oxidosqualene cyclases from Stigmatella ... | 33–676 | 1.09e-49 |
| PLN02993 | PLN02993 | lupeol synthase | 18–566 | 6.57e-33 |
| PLN03012 | PLN03012 | Camelliol C synthase | 24–675 | 9.97e-33 |
| Prenyltrans_2 | pfam13249 | Prenyltransferase-like; | 431–562 | 4.07e-16 |
| Prenyltrans_1 | pfam13243 | Prenyltransferase-like; | 495–614 | 2.09e-14 |

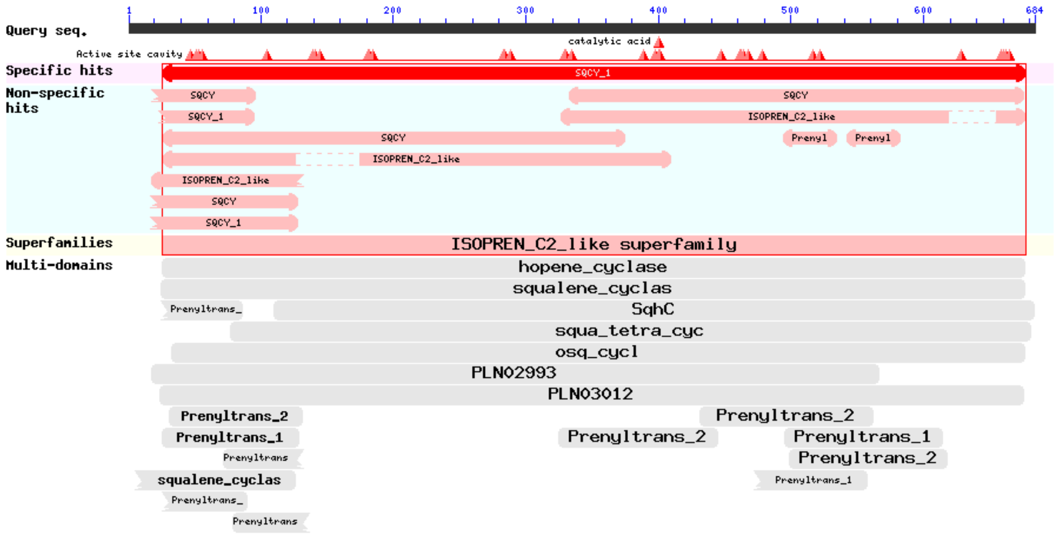


**Figure S16.** Conserved domains detected in the squalene-hopene/tetraprenyl-beta-curcumene cyclase gene from *Bradyrhizobium sp* (tr|A4Z167 A4Z167_BRASO) (edited from the NCBI CDD database, [1]).

**Table S17.** List of domain hits for a putative lupeol synthase 1 gene from *L. dendroidea*.

| **Name** | **Accession** | **Description** | **Interval** | **E-Value** |
|---|---|---|---|---|
| SQCY_1 | cd02892 | Squalene cyclase (SQCY) domain subgroup 1; found in class II terpene cyclases that have an ... | 1363 | 5.98e-143 |
| SQCY | cd02889 | Squalene cyclase (SQCY) domain; found in class II terpene cyclases that have an alpha 6 - ... | 24–363 | 8.74e-126 |
| ISOPREN_C2_like | cd00688 | This group contains class II terpene cyclases, protein prenyltransferases beta subunit, two ... | 1–363 | 6.47e-39 |
| Prenyltrans | pfam00432 | Prenyltransferase and squalene oxidase repeat; | 207–240 | 3.26e-04 |
| SQCY_1 | cd02892 | Squalene cyclase (SQCY) domain subgroup 1; found in class II terpene cyclases that have an ... | 207–274 | 4.34e-03 |
| Prenyltrans_2 | pfam13249 | Prenyltransferase-like; | 211–331 | 7.97e-09 |
| Prenyltrans_2 | pfam13249 | Prenyltransferase-like; | 134–275 | 5.74e-08 |
| squalene_cyclas | TIGR01787 | squalene/oxidosqualene cyclases; This family of enzymes catalyzes the cyclization of the ... | 3–363 | 1.19e-117 |
| PLN03012 | PLN03012 | Camelliol C synthase | 3–363 | 1.41e-106 |
| PLN02993 | PLN02993 | lupeol synthase | 3–369 | 4.48e-97 |
| osq_cycl | TIGR03463 | 2,3-oxidosqualene cyclase; This model identifies 2,3-oxidosqualene cyclases from Stigmatella ... | 1–361 | 1.37e-91 |
| SqhC | COG1657 | Squalene cyclase [Lipid metabolism] | 3–367 | 4.95e-56 |

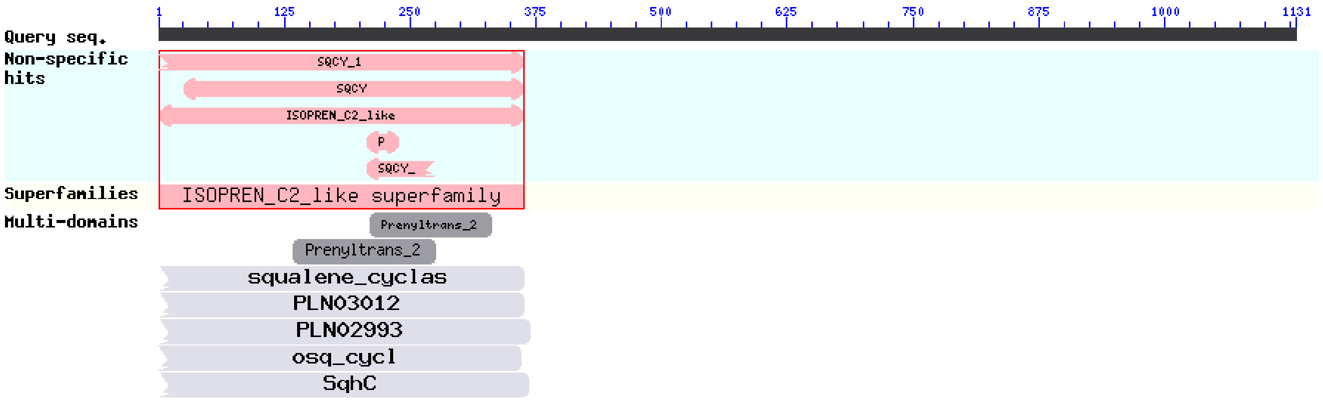


**Figure S17.** Conserved domains detected in a putative lupeol synthase 1 gene from *L. dendroidea* (edited from the NCBI CDD database, [1]).

**Table S18.** List of domain hits for the lupeol synthase 1 gene from *Arabidopsis thaliana* (sp|Q9C5M3|LUP1_ARATH).

| Name | Accession | Description | Interval | E-Value |
|---|---|---|---|---|
| SQCY_1 | cd02892 | Squalene cyclase (SQCY) domain subgroup 1; found in class II terpene cyclases that have an ... | 99–749 | 0e+00 |
| Prenyltrans | pfam00432 | Prenyltransferase and squalene oxidase repeat; | 589–629 | 1.24e-07 |
| PLN02993 | PLN02993 | lupeol synthase | 1–757 | 0e+00 |
| squalene_cyclas | TIGR01787 | squalene/oxidosqualene cyclases; This family of enzymes catalyzes the cyclization of the ... | 98–751 | 0e+00 |
| SqhC | COG1657 | Squalene cyclase [Lipid metabolism] | 186–749 | 3.47e-59 |
| Prenyltrans_2 | pfam13249 | Prenyltransferase-like | 593–717 | 5.37e-15 |

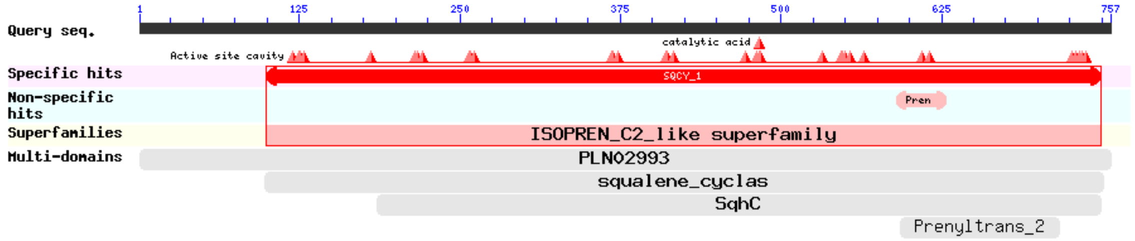


**Figure S18.** Conserved domains detected in the lupeol synthase 1 gene from *Arabidopsis thaliana* (sp|Q9C5M3|LUP1_ARATH) (edited from the NCBI CDD database, [1]).

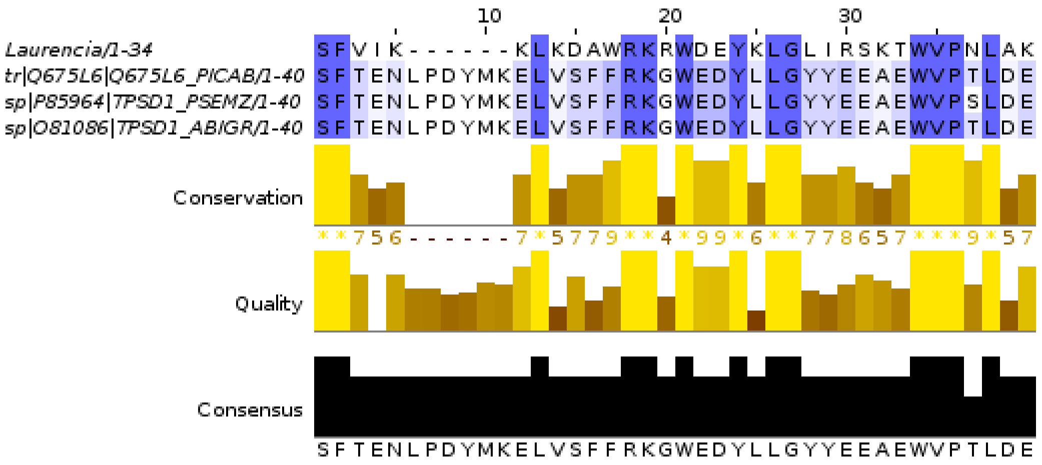


**Figure S19.** Multiple alignment analysis between a putative alpha-bisabolene synthase from *L. dendroidea* and alpha-bisabolene synthase reference sequences from *Picea abies*, *Pseudotsuga menziesii* and *Abies grandis* downloaded from Uniprot. The alignment was done using Muscle software [2] and visualized with Jalview [3].

**Table S19.** List of domain hits for the alpha-bisabolene synthase gene from *Picea abies* (tr|Q675L6 Q675L6_PICAB).

| Name | Accession | Description | Interval | E-Value |
|---|---|---|---|---|
| Terpene_cyclase_plant_C1 | cd00684 | Plant Terpene Cyclases, Class 1; This CD includes a diverse group of monomeric plant terpene ... | 271–804 | 0e+00 |
| Terpene_synth_C | pfam03936 | Terpene synthase family, metal binding domain; It has been suggested that this gene family be ... | 481–751 | 1.09e-97 |
| Terpene_cyclase_C1 | cd00868 | Terpene cyclases, Class 1; Terpene cyclases, Class 1 (C1) of the class 1 family of isoprenoid ... | 495–775 | 3.12e-89 |
| Terpene_synth | pfam01397 | Terpene synthase, *N*-terminal domain; It has been suggested that this gene family be designated ... | 246–439 | 6.37e-66 |
| Isoprenoid_Biosyn_C1 | cd00385 | Isoprenoid Biosynthesis enzymes, Class 1; Superfamily of trans-isoprenyl diphosphate synthases ... | 534–771 | 1.28e-29 |
| PLN02150 | PLN02150 | terpene synthase/cyclase family protein | 714–807 | 1.15e-07 |
| ISOPREN_C2_like | cd00688 | This group contains class II terpene cyclases, protein prenyltransferases beta subunit, two ... | 73–156 | 3.94e-03 |
| PLN02279 | PLN02279 | ent-kaur-16-ene synthase | 60–807 | 5.06e-148 |
| PLN02592 | PLN02592 | ent-copalyl diphosphate synthase | 57–713 | 4.31e-165 |
| Prenyltrans_1 | pfam13243 | Prenyltransferase-like; | 99–157 | 9.07e-04 |
| Prenyltrans_1 | pfam13243 | Prenyltransferase-like; | 78-154 | 1.33e-03 |

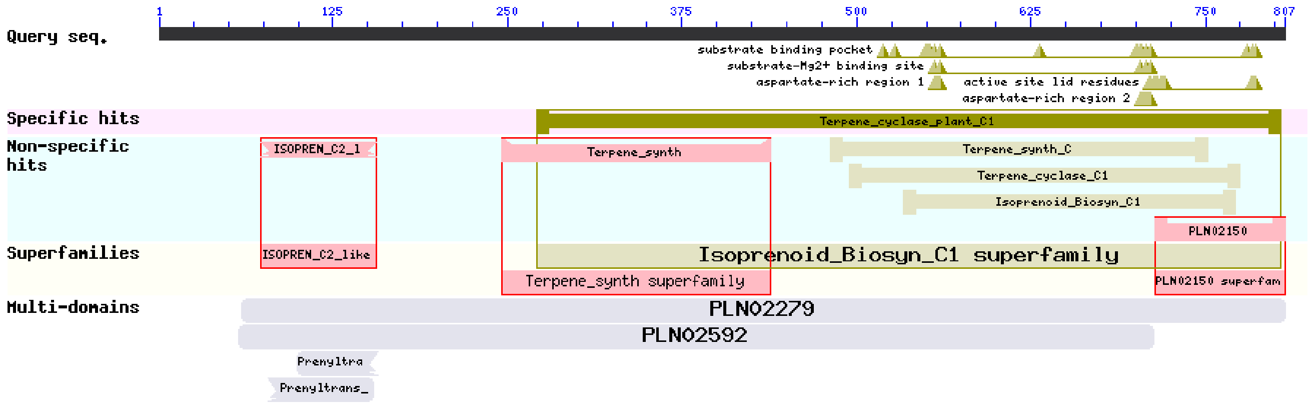


**Figure S20.** Conserved domains detected in the alpha-bisabolene synthase gene from *Picea abies* (tr|Q675L6 Q675L6_PICAB) (edited from the NCBI CDD database, [1]).

**Table S20.** List of domain hits for a putative germacrene-*A* synthase gene from *L. dendroidea*.

| **Name** | **Accession** | **Description** | **Interval** | **E-Value** |
|---|---|---|---|---|
| Terpene_cyclase_nonplant_C1 | cd00687 | Non-plant Terpene Cyclases, Class 1; This CD includes terpenoid cyclases such as pentalenene ... | 56-353 | 8.36e-25 |
| Terpene_cyclase_C1 | cd00868 | Terpene cyclases, Class 1; Terpene cyclases, Class 1 (C1) of the class 1 family of isoprenoid ... | 60-349 | 2.45e-11 |
| Terpene_synth_C | pfam03936 | Terpene synthase family, metal binding domain; It has been suggested that this gene family be ... | 56-314 | 1.07e-04 |

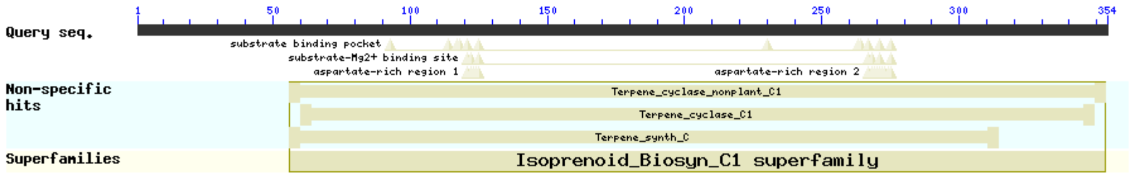


**Figure S21.** Conserved domains detected in a putative germacrene-*A* synthase gene from *L. dendroidea* (edited from the NCBI CDD database, [1]).

**Table S21.** List of domain hits for the germacrene-*A* synthase gene from *Nostoc punctiforme* (sp|B2J4A4 GERAS_NOSP7).

| **Name** | **Accession** | **Description** | **Interval** | **E-Value** |
|---|---|---|---|---|
| Terpene_cyclase_nonplant_C1 | cd00687 | Non-plant Terpene Cyclases, Class 1; This CD includes terpenoid cyclases such as pentalenene ... | 10–309 | 3.27e-123 |
| Terpene_cyclase_C1 | cd00868 | Terpene cyclases, Class 1; Terpene cyclases, Class 1 (C1) of the class 1 family of isoprenoid ... | 23–305 | 7.93e-51 |
| Terpene_synth_C | pfam03936 | Terpene synthase family, metal binding domain; It has been suggested that this gene family be ... | 31–264 | 1.87e-20 |

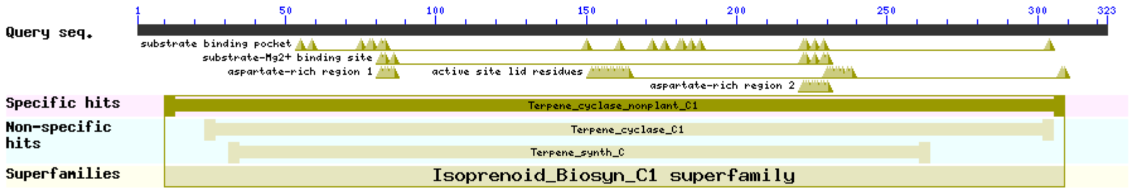


**Figure S22.** Conserved domains detected in the germacrene-A synthase gene from *Nostoc punctiforme* (sp|B2J4A4 GERAS_NOSP7) (edited from the NCBI CDD database, [1]).

**Table S22.** List of domain hits for a putative germacrene *A* oxidase gene from *L. dendroidea*.

| Name | Accession | Description | Interval | E-Value |
|---|---|---|---|---|
| p450 | pfam00067 | Cytochrome P450; Cytochrome P450s are haem-thiolate proteins involved in the oxidative ... | 367–564 | 1.03e-44 |
| CypX | COG2124 | Cytochrome P450 [Secondary metabolites biosynthesis, transport, and catabolism] | 367–535 | 3.07e-31 |
| PLN02687 | PLN02687 | flavonoid 3′-monooxygenase | 370–531 | 6.05e-21 |
| PTZ00404 | PTZ00404 | cytochrome P450; Provisional | 370–539 | 3.35e-18 |
| PLN02738 | PLN02738 | carotene beta-ring hydroxylase | 370–534 | 1.21e-17 |

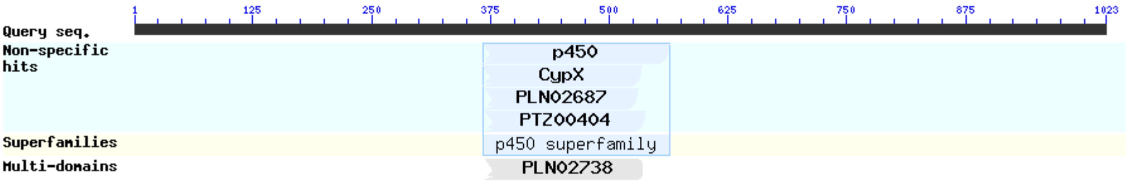


**Figure S23.** Conserved domains detected in a putative germacrene *A* oxidase gene from *L. dendroidea* (edited from the NCBI CDD database [1]).

**Table S23.** List of domain hits for the germacrene *A* oxidase gene from *Cichorium intybus* (sp|D5JBW8|GAO_CICIN).

| Name | Accession | Description | Interval | E-value |
|---|---|---|---|---|
| PLN02687 | PLN02687 | flavonoid 3′-monooxygenase | 5–486 | 4.19e-134 |
| p450 | pfam00067 | Cytochrome P450; Cytochrome P450s are haem-thiolate proteins involved in the oxidative ... | 33–460 | 7.76e-99 |
| PTZ00404 | PTZ00404 | cytochrome P450; Provisional | 5–477 | 1.31e-56 |
| CypX | COG2124 | Cytochrome P450 [Secondary metabolites biosynthesis, transport, and catabolism] | 67–460 | 1.34e-38 |
| PLN02738 | PLN02738 | carotene beta-ring hydroxylase | 31–487 | 6.67e-29 |

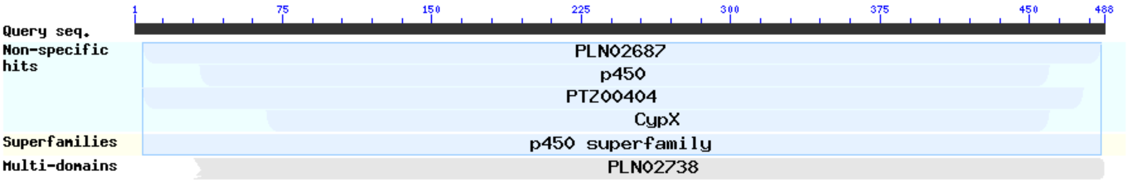


**Figure S24.** Conserved domains detected in the germacrene *A* oxidase gene from *Cichorium intybus* (sp|D5JBW8|GAO_CICIN) (edited from the NCBI CDD database, [1].

**Table S24.** List of domain hits for a putative aristolochene synthase gene from *L. dendroidea*.

| Name | Accession | Description | Interval | E-Value |
|---|---|---|---|---|
| Terpene_cyclase_nonplant_C1 | cd00687 | Non-plant Terpene Cyclases, Class 1; This CD includes terpenoid cyclases such as pentalenene | 56–353 | 8.36e-25 |
| Terpene_cyclase_C1 | cd00868 | Terpene cyclases, Class 1; Terpene cyclases, Class 1 (C1) of the class 1 family of isoprenoid | 60–349 | 2.45e-11 |
| Terpene_synth_C | pfam03936 | Terpene synthase family, metal binding domain; It has been suggested that this gene family be | 56–314 | 1.07e-04 |

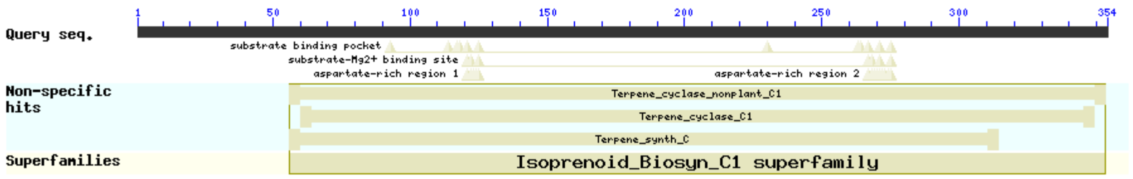


**Figure S25.** Conserved domains detected in a putative aristolochene synthase gene from *L. dendroidea* (edited from the NCBI CDD database, [1]).

**Table S25.** List of domain hits for the aristolochene synthase gene from *Aspergillus terreus* (sp|Q9UR08 ARIS_ASPTE).

| Name | Accession | Description | Interval | E-Value |
|---|---|---|---|---|
| Terpene_cyclase_nonplant_C1 | cd00687 | Non-plant Terpene Cyclases, Class 1; This CD includes terpenoid cyclases such as pentalenene | 18–312 | 1.26e-98 |
| Terpene_cyclase_C1 | cd00868 | Terpene cyclases, Class 1; Terpene cyclases, Class 1 (C1) of the class 1 family of isoprenoid | 28–308 | 6.00e-32 |
| Terpene_synth_C | pfam03936 | Terpene synthase family, metal binding domain; It has been suggested that this gene family be | 21–269 | 2.55e-30 |
| Isoprenoid_Biosyn_C1 | cd00385 | Isoprenoid Biosynthesis enzymes, Class 1; Superfamily of trans-isoprenyl diphosphate synthases | 63–280 | 6.29e-09 |

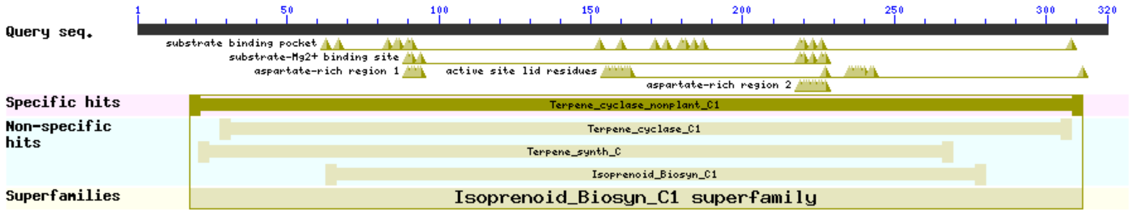


**Figure S26.** Conserved domains detected in the aristolochene synthase gene from *Aspergillus terreus* (sp|Q9UR08 ARIS_ASPTE) (edited from the NCBI CDD database, [1]).

**Table S26.** List of domain hits for a putative 5-epiaristolochene 1,3-dihydroxylase gene from *L. dendroidea*.

| **Name** | **Accession** | **Description** | **Interval** | **E-Value** |
|---|---|---|---|---|
| p450 | pfam00067 | Cytochrome P450; Cytochrome P450s are haem-thiolate proteins involved in the oxidative ... | 367–564 | 1.10e-44 |
| CypX | COG2124 | Cytochrome P450 [Secondary metabolites biosynthesis, transport, and catabolism] | 367–535 | 3.30e-31 |
| PLN02687 | PLN02687 | flavonoid 3′-monooxygenase | 370–531 | 5.92e-21 |
| PLN02183 | PLN02183 | ferulate 5-hydroxylase | 367–530 | 2.79e-20 |
| PLN02302 | PLN02302 | ent-kaurenoic acid oxidase | 384–551 | 1.27e-19 |
| PLN02966 | PLN02966 | cytochrome P450 83A1 | 367–548 | 3.18e-19 |
| PLN02394 | PLN02394 | trans-cinnamate 4-monooxygenase | 373–521 | 8.60e-19 |
| PLN02655 | PLN02655 | ent-kaurene oxidase | 371–517 | 2.94e-18 |
| PTZ00404 | PTZ00404 | cytochrome P450; Provisional | 370–539 | 3.54e-18 |
| PLN03195 | PLN03195 | fatty acid omega-hydroxylase; Provisional | 367–529 | 1.14e-17 |
| PLN03234 | PLN03234 | cytochrome P450 83B1; Provisional | 367–526 | 1.40e-16 |
| PLN02290 | PLN02290 | cytokinin trans-hydroxylase | 373–540 | 5.60e-16 |
| PLN02936 | PLN02936 | epsilon-ring hydroxylase | 370–529 | 6.75e-16 |
| PLN02196 | PLN02196 | abscisic acid 8′-hydroxylase | 367–538 | 1.04e-15 |
| PLN00110 | PLN00110 | flavonoid 3′,5′-hydroxylase (F3′5′H); Provisional | 367–521 | 1.60e-15 |
| PLN02169 | PLN02169 | fatty acid (omega-1)-hydroxylase/midchain alkane hydroxylase | 367–537 | 4.63e-15 |
| PLN02426 | PLN02426 | cytochrome P450, family 94, subfamily C protein | 367–545 | 6.97e-15 |
| PLN02500 | PLN02500 | cytochrome P450 90B1 | 413–536 | 3.43e-14 |
| PLN03112 | PLN03112 | cytochrome P450 family protein; Provisional | 370–524 | 9.26e-13 |
| PLN02987 | PLN02987 | Cytochrome P450, family 90, subfamily A | 367–536 | 1.95e-12 |
| PLN03141 | PLN03141 | 3-epi-6-deoxocathasterone 23-monooxygenase; Provisional | 365–535 | 4.81e-09 |
| PLN02774 | PLN02774 | brassinosteroid-6-oxidase | 367–524 | 5.38e-09 |
| PLN00168 | PLN00168 | Cytochrome P450; Provisional | 371–525 | 2.91e-08 |
| PLN03018 | PLN03018 | homomethionine *N*-hydroxylase | 384–520 | 4.02e-08 |
| PLN02971 | PLN02971 | tryptophan *N*-hydroxylase | 370–522 | 4.17e-08 |
| PLN02648 | PLN02648 | allene oxide synthase | 412–489 | 8.49e-08 |
| PLN02738 | PLN02738 | carotene beta-ring hydroxylase | 370–534 | 1.28e-17 |

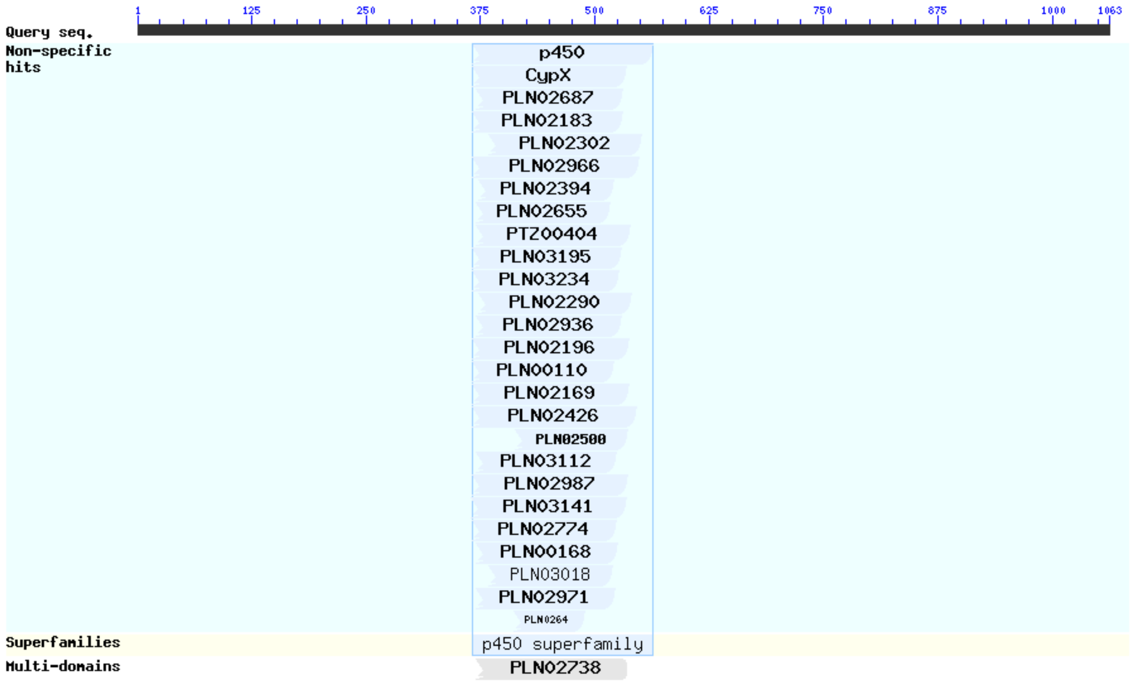


**Figure S27.** Conserved domains detected in a putative 5-epiaristolochene 1,3-dihydroxylase gene from *L. dendroidea* (edited from the NCBI CDD database, [1]).

**Table S27.** List of domain hits for the 5-epiaristolochene 1,3-dihydroxylase gene from *Nicotiana tabacum* (sp|Q94FM7|C71DK_TOBAC).

| Name | Accession | Description | Interval | E-Value |
|---|---|---|---|---|
| PLN02183 | PLN02183 | ferulate 5-hydroxylase | 33–488 | 9.91e-128 |
| PLN02687 | PLN02687 | flavonoid 3′-monooxygenase | 30–498 | 5.87e-123 |
| PLN03112 | PLN03112 | cytochrome P450 family protein; Provisional | 4–498 | 6.11e-114 |
| p450 | pfam00067 | Cytochrome P450; Cytochrome P450s are haem-thiolate proteins involved in the oxidative ... | 33–494 | 2.34e-109 |
| PLN03234 | PLN03234 | cytochrome P450 83B1; Provisional | 23–499 | 4.35e-109 |
| PLN02966 | PLN02966 | cytochrome P450 83A1 | 22–494 | 2.38e-103 |
| PLN00110 | PLN00110 | flavonoid 3′,5′-hydroxylase (F3′5′H); Provisional | 29–498 | 2.76e-102 |
| PLN02394 | PLN02394 | trans-cinnamate 4-monooxygenase | 31–483 | 1.57e-81 |
| PLN02971 | PLN02971 | tryptophan *N*-hydroxylase | 25–468 | 5.45e-60 |
| PLN02655 | PLN02655 | ent-kaurene oxidase | 34–498 | 8.16e-59 |
| PLN00168 | PLN00168 | Cytochrome P450; Provisional | 20–466 | 4.94e-57 |
| PLN03018 | PLN03018 | homomethionine *N*-hydroxylase | 25–477 | 8.67e-54 |
| PTZ00404 | PTZ00404 | cytochrome P450; Provisional | 24–486 | 1.12e-53 |
| CypX | COG2124 | Cytochrome P450 [Secondary metabolites biosynthesis, transport, and catabolism] | 33–470 | 4.01e-43 |
| PLN02936 | PLN02936 | epsilon-ring hydroxylase | 62–497 | 5.84e-36 |
| PLN02302 | PLN02302 | ent-kaurenoic acid oxidase | 234–463 | 4.10e-28 |
| PLN03195 | PLN03195 | fatty acid omega-hydroxylase; Provisional | 27–463 | 5.47e-27 |

**Table S27.** Cont.

| | | | | |
|---|---|---|---|---|
| PLN02290 | PLN02290 | cytokinin trans-hydroxylase | 219–463 | 1.48e-25 |
| PLN02987 | PLN02987 | Cytochrome P450, family 90, subfamily A | 31–465 | 2.75e-23 |
| PLN02196 | PLN02196 | abscisic acid 8′-hydroxylase | 3–467 | 2.78e-21 |
| PLN02774 | PLN02774 | brassinosteroid-6-oxidase | 233–463 | 1.42e-20 |
| PLN02169 | PLN02169 | fatty acid (omega-1)-hydroxylase/midchain alkane hydroxylase | 230–470 | 3.80e-20 |
| PLN03141 | PLN03141 | 3-epi-6-deoxocathasterone 23-monooxygenase; Provisional | 234–465 | 1.83e-18 |
| PLN02426 | PLN02426 | cytochrome P450, family 94, subfamily C protein | 122–444 | 3.98e-15 |
| PLN02500 | PLN02500 | cytochrome P450 90B1 | 233–467 | 2.22e-14 |
| PLN02648 | PLN02648 | allene oxide synthase | 331–418 | 7.72e-06 |
| PLN02738 | PLN02738 | carotene beta-ring hydroxylase | 269–497 | 3.46e-34 |

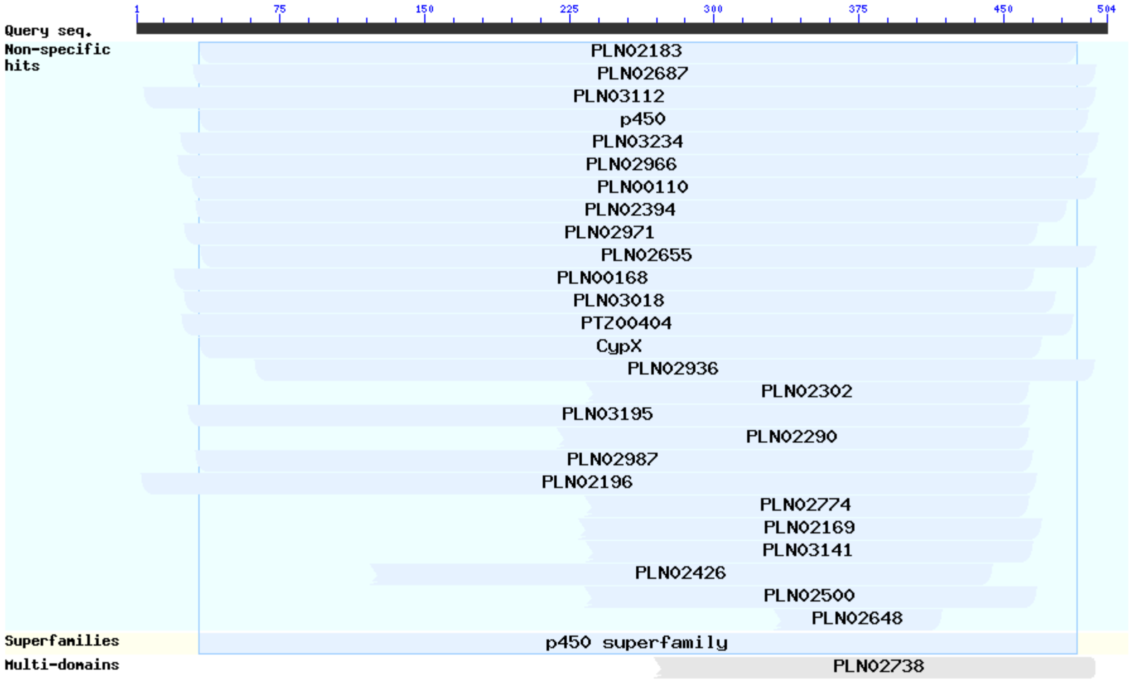


**Figure S28.** Conserved domains detected in the 5-epiaristolochene 1,3-dihydroxylase gene from *Nicotiana tabacum* (sp|Q94FM7|C71DK_TOBAC) (edited from the NCBI CDD database, [1]).

**Table S28.** List of domain hits for a putative pentalenene synthase gene from *L. dendroidea*.

| Name | Accession | Description | Interval | E-value |
|---|---|---|---|---|
| Terpene_cyclase_nonplant_C1 | cd00687 | Non-plant Terpene Cyclases, Class 1; This CD includes terpenoid cyclases such as pentalenene ... | 11–298 | 3.90e-21 |
| Terpene_cyclase_C1 | cd00868 | Terpene cyclases, Class 1; Terpene cyclases, Class 1 (C1) of the class 1 family of isoprenoid ... | 15–282 | 4.15e-09 |
| Terpene_synth_C | pfam03936 | Terpene synthase family, metal binding domain; It has been suggested that this gene family be ... | 15–262 | 2.96e-04 |

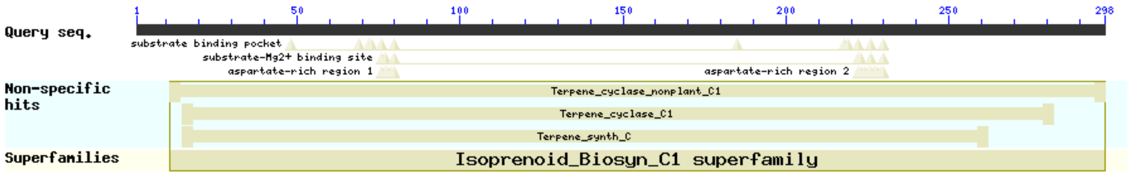


**Figure S29.** Conserved domains detected in a putative pentalenene synthase gene from *L. dendroidea* (edited from the NCBI CDD database, [1]).

**Table S29.** List of domain hits for the pentalenene synthase gene from *Streptomyces exfoliatus* (sp|Q55012 PENA_STREX).

| Name | Accession | Description | Interval | E-Value |
|---|---|---|---|---|
| Terpene_cyclase_nonplant_C1 | cd00687 | Non-plant Terpene Cyclases, Class 1; This CD includes terpenoid cyclases such as pentalenene ... | 8–313 | 2.37e-108 |
| Terpene_cyclase_C1 | cd00868 | Terpene cyclases, Class 1; Terpene cyclases, Class 1 (C1) of the class 1 family of isoprenoid ... | 19–309 | 4.49e-41 |
| Terpene_synth_C | pfam03936 | Terpene synthase family, metal binding domain; It has been suggested that this gene family be ... | 29–265 | 1.57e-34 |
| Isoprenoid_Biosyn_C1 | cd00385 | Isoprenoid Biosynthesis enzymes, Class 1; Superfamily of trans-isoprenyl diphosphate synthases ... | 72–292 | 9.90e-10 |

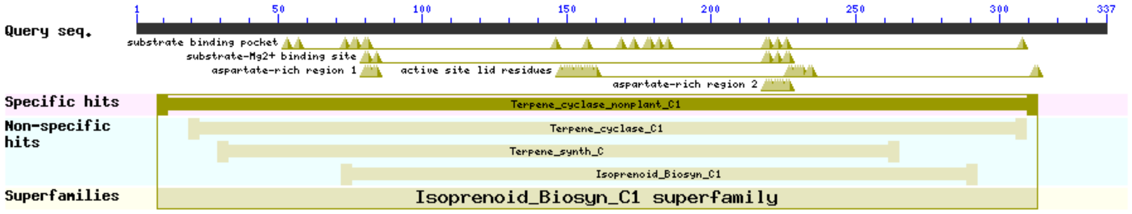


**Figure S30.** Conserved domains detected in the pentalenene synthase gene from *Streptomyces exfoliatus* (sp|Q55012 PENA_STREX) (edited from the NCBI CDD database, [1]).

**Table S30.** List of domain hits for a putative zerumbone synthase gene from *L. dendroidea*.

| Name | Accession | Description | Interval | E-Value |
|---|---|---|---|---|
| SDR_c | cd05233 | classical (c) SDRs; SDRs are a functionally diverse family of oxidoreductases that have a ... | 44–275 | 1.22e-32 |
| fabG | PRK06550 | 3-ketoacyl-(acyl-carrier-protein) reductase; Provisional | 41–233 | 4.23e-17 |
| adh_short_C2 | pfam13561 | Enoyl-(Acyl carrier protein) reductase; | 51–278 | 1.00e-07 |

**Table S30.** *Cont.*

| | | | | |
|---|---|---|---|---|
| PKS_KR | smart00822 | This enzymatic domain is part of bacterial polyketide synthases; It catalyses the first step ... | 42–132 | 1.88e-05 |
| FabG | COG1028 | Dehydrogenases with different specificities (related to short-chain alcohol dehydrogenases) ... | 39–278 | 2.19e-34 |
| fabG | PRK05565 | 3-ketoacyl-(acyl-carrier-protein) reductase; Provisional | 38–242 | 7.99e-26 |
| PHB_DH | TIGR01963 | 3-hydroxybutyrate dehydrogenase; This model represents a subfamily of the short chain ... | 41–279 | 1.08e-22 |
| adh_short | pfam00106 | short chain dehydrogenase; This family contains a wide variety of dehydrogenases. | 42–209 | 1.95e-16 |
| PLN02253 | PLN02253 | xanthoxin dehydrogenase | 36–224 | 2.44e-13 |
| secoisolariciresinol-DH_like_SDR_c | cd05326 | secoisolariciresinol dehydrogenase (secoisolariciresinol-DH)-like, classical (c) SDRs; | 38–225 | 1.86e-15 |

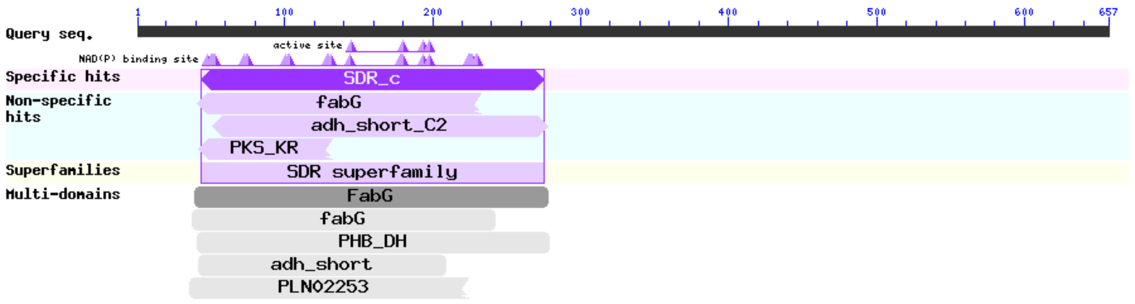


**Figure S31.** Conserved domains detected in a putative zerumbone synthase gene from *L. dendroidea* (edited from the NCBI CDD database, [1]).

**Table S31.** List of domain hits for the zerumbone synthase gene from *Zingiber zerumbet* (sp|F1SWA0|ZERSY_ZINZE).

| Name | Accession | Description | Interval | E-Value |
|---|---|---|---|---|
| secoisolariciresinol-DH_like_SDR_c | cd05326 | secoisolariciresinol dehydrogenase (secoisolariciresinol-DH)-like, classical (c) SDRs; ... | 2–257 | 5.98e-118 |
| PRK07069 | PRK07069 | short chain dehydrogenase; Validated | 8–257 | 5.07e-52 |
| adh_short_C2 | pfam13561 | Enoyl-(Acyl carrier protein) reductase; | 12–257 | 2.29e-27 |
| PKS_KR | smart00822 | This enzymatic domain is part of bacterial polyketide synthases; It catalyses the first step ... | 6–151 | 2.29e-08 |
| PLN02730 | PLN02730 | enoyl-[acyl-carrier-protein] reductase | 122–255 | 1.58e-04 |

**Table S31.** *Cont.*

| | | | | |
|---|---|---|---|---|
| PLN02253 | PLN02253 | xanthoxin dehydrogenase | 2–267 | 1.11e-165 |
| fabG | PRK07231 | 3-ketoacyl-(acyl-carrier-protein) reductase; Provisional | 1–258 | 4.45e-76 |
| FabG | COG1028 | Dehydrogenases with different specificities (related to short-chain alcohol dehydrogenases) ... | 1–257 | 9.62e-59 |
| 3oxo_ACP_reduc | TIGR0183 0 | 3-oxoacyl-(acyl-carrier-protein) reductase; This model represents 3-oxoacyl-[ACP] reductase, ... | 8–255 | 2.40e-55 |
| adh_short | pfam00106 | short chain dehydrogenase; This family contains a wide variety of dehydrogenases. | 6–173 | 4.49e-30 |

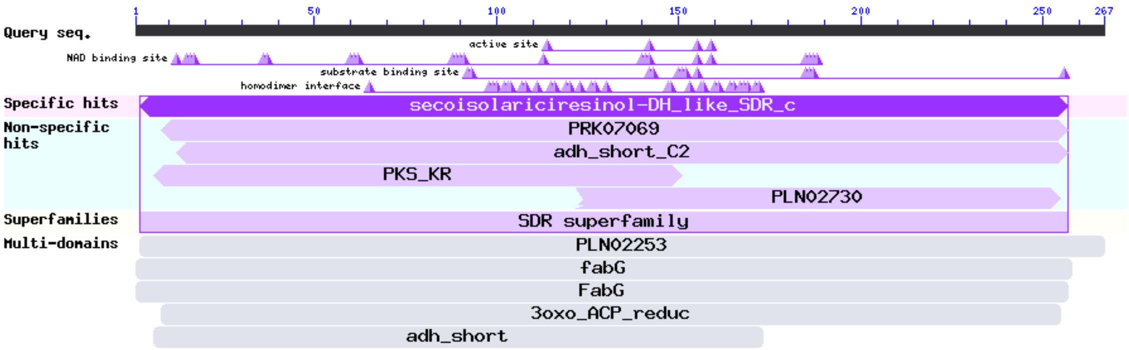


**Figure S32.** Conserved domains detected in the zerumbone synthase gene from *Zingiber zerumbet* (sp|F1SWA0|ZERSY_ZINZE) (edited from the NCBI CDD database, [1]).

**Table S32.** List of domain hits for a putative gibberelin 20-oxidase gene from *L. dendroidea*.

| Name | Accession | Description | Interval | E-Value |
|---|---|---|---|---|
| DIOX_N | pfam14226 | non-haem dioxygenase in morphine synthesis *N*-terminal; This is the highly conserved *N*-terminal ... | 2129–2401 | 2.84e-12 |
| 2OG-FeII_Oxy | pfam03171 | 2OG-Fe(II) oxygenase superfamily; This family contains members of the 2-oxoglutarate (2OG) and ... | 1616–1954 | 7.56e-09 |
| PTZ00273 | PTZ00273 | oxidase reductase; Provisional | 1508–2443 | 8.96e-24 |
| PcbC | COG3491 | Isopenicillin N synthase and related dioxygenases [General function prediction only] | 1595–2419 | 5.94e-29 |
| PLN02485 | PLN02485 | Oxidoreductase | 1511–2497 | 2.50e-19 |
| PLN03002 | PLN03002 | oxidoreductase, 2OG-Fe(II) oxygenase family protein | 1508–2362 | 6.26e-18 |
| PLN02750 | PLN02750 | oxidoreductase, 2OG-Fe(II) oxygenase family protein | 1517–2443 | 1.70e-12 |
| PLN02393 | PLN02393 | leucoanthocyanidin dioxygenase like protein | 1529–2521 | 1.25e-11 |
| PLN02276 | PLN02276 | gibberellin 20-oxidase | 1598–2497 | 1.94e-10 |

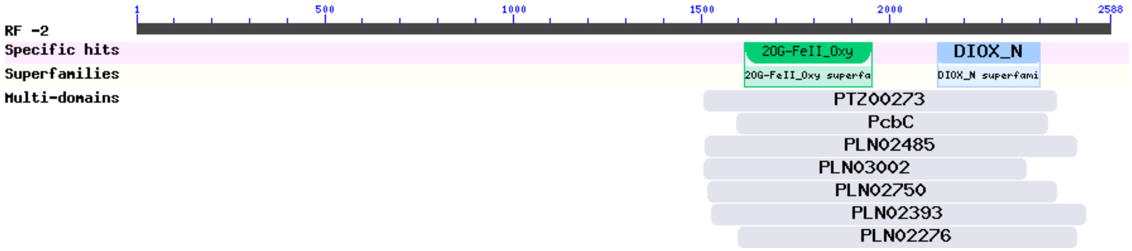


**Figure S33.** Conserved domains detected in a putative gibberelin 20-oxidase gene from *L. dendroidea* (edited from the NCBI CDD database, [1]).

**Table S33.** List of domain hits for the gibberelin 20-oxidase gene from *Arabidopsis thaliana* (gi|60390168|sp|Q39112.1|GAOX3_ARATH).

| Name | Accession | Description | Interval | E-value |
|---|---|---|---|---|
| 2OG-FeII_Oxy | pfam03171 | 2OG-Fe(II) oxygenase superfamily; This family contains members of the 2-oxoglutarate (2OG) and ... | 222–321 | 3.06e-39 |
| DIOX_N | pfam14226 | non-haem dioxygenase in morphine synthesis *N*-terminal; This is the highly conserved *N*-terminal ... | 58–160 | 3.86e-21 |
| PLN03176 | PLN03176 | flavanone-3-hydroxylase; Provisional | 41–137 | 1.49e-05 |
| PLN02276 | PLN02276 | gibberellin 20-oxidase | 17–380 | 0e+00 |
| PLN02393 | PLN02393 | leucoanthocyanidin dioxygenase like protein | 37–334 | 9.01e-65 |
| PLN02254 | PLN02254 | gibberellin 3-beta-dioxygenase | 22–360 | 7.45e-62 |
| PLN03178 | PLN03178 | leucoanthocyanidin dioxygenase; Provisional | 37–354 | 5.59e-61 |
| PLN02912 | PLN02912 | oxidoreductase, 2OG-Fe(II) oxygenase family protein | 36–370 | 4.36e-60 |
| PLN02639 | PLN02639 | oxidoreductase, 2OG-Fe(II) oxygenase family protein | 26–350 | 4.80e-58 |
| PLN02758 | PLN02758 | oxidoreductase, 2OG-Fe(II) oxygenase family protein | 37–360 | 6.27e-57 |
| PLN02750 | PLN02750 | oxidoreductase, 2OG-Fe(II) oxygenase family protein | 37–353 | 3.35e-53 |
| PcbC | COG3491 | Isopenicillin *N* synthase and related dioxygenases [General function prediction only] | 54–361 | 5.21e-52 |

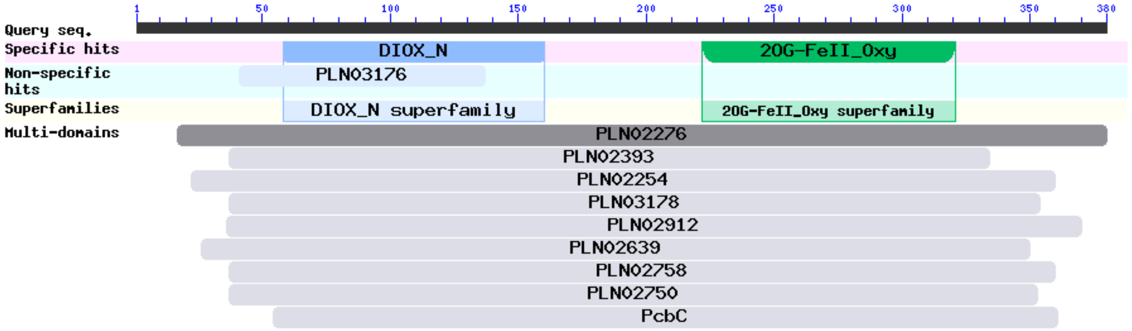


**Figure S34.** Conserved domains detected in the gibberelin 20-oxidase gene from *Arabidopsis thaliana* (gi|60390168|sp|Q39112.1|GAOX3_ARATH) (edited from the NCBI CDD database, [1]).

**Table S34.** List of domain hits for a putative gibberelin 2-oxidase gene from *L. dendroidea*.

| Name | Accession | Description | Interval | E-Value |
|---|---|---|---|---|
| DIOX_N | pfam14226 | non-haem dioxygenase in morphine synthesis *N*-terminal; This is the highly conserved *N*-terminal ... | 97–471 | 4.75e-09 |
| 2OG-FeII_Oxy | pfam03171 | 2OG-Fe(II) oxygenase superfamily; This family contains members of the 2-oxoglutarate (2OG) and ... | 610–927 | 4.13e-07 |
| PTZ00273 | PTZ00273 | oxidase reductase; Provisional | 94–1035 | 1.91e-32 |
| PcbC | COG3491 | Isopenicillin *N* synthase and related dioxygenases [General function prediction only] | 85–948 | 1.05e-32 |
| PLN02485 | PLN02485 | oxidoreductase | 91–873 | 1.63e-15 |
| PLN03002 | PLN03002 | oxidoreductase, 2OG-Fe(II) oxygenase family protein | 214–1056 | 2.60e-09 |
| PLN02156 | PLN02156 | gibberellin 2-beta-dioxygenase | 79–873 | 3.20e-08 |

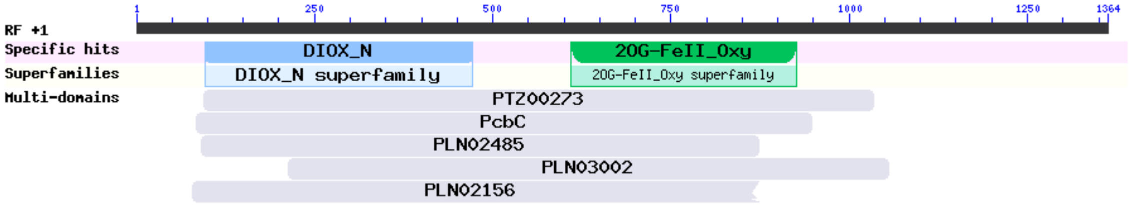


**Figure S35.** Conserved domains detected in a putative gibberelin 2-oxidase gene from *L. dendroidea* (edited from the NCBI CDD database, [1]).

**Table S35.** List of domain hits for the the gibberelin 2-oxidase gene from *Arabidopsis thaliana*.

| Name | Accession | Description | Interval | E-Value |
|---|---|---|---|---|
| 2OG-FeII_Oxy | pfam03171 | 2OG-Fe(II) oxygenase superfamily; This family contains members of the 2-oxoglutarate (2OG) and ... | 157–269 | 3.98e-28 |
| DIOX_N | pfam14226 | non-haem dioxygenase in morphine synthesis *N*-terminal; This is the highly conserved *N*-terminal ... | 15–108 | 9.31e-20 |
| PLN03176 | PLN03176 | flavanone-3-hydroxylase; Provisional | 9–71 | 3.40e-09 |
| PcbC | COG3491 | Isopenicillin *N* synthase and related dioxygenases [General function prediction only] | 13–309 | 1.14e-49 |
| PLN02156 | PLN02156 | gibberellin 2-beta-dioxygenase | 15–316 | 7.18e-98 |

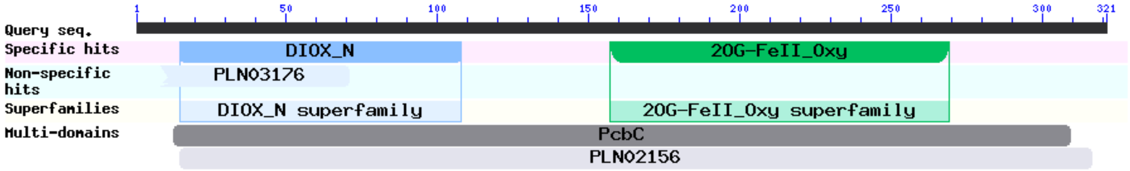


**Figure S36.** Conserved domains detected in the gibberelin 2-oxidase gene from *Arabidopsis thaliana* (gi|75308865|sp|Q9C7Z1.1|G2OX4_ARATH) (edited from the NCBI CDD database, [1]).

**Table S36.** List of domain hits for a putative abietadienol/ abietadienal oxidase gene from *L. dendroidea*.

| Name | Accession | Description | Interval | E-Value |
|---|---|---|---|---|
| PLN02936 | PLN02936 | epsilon-ring hydroxylase | 135–620 | 5.82e-141 |
| p450 | pfam00067 | Cytochrome P450; Cytochrome P450s are haem-thiolate proteins involved in the oxidative ... | 139–613 | 6.45e-78 |
| CypX | COG2124 | Cytochrome P450 [Secondary metabolites biosynthesis, transport, and catabolism] | 154–620 | 2.27e-42 |
| PLN02290 | PLN02290 | cytokinin trans-hydroxylase | 207–618 | 1.36e-32 |
| PLN03195 | PLN03195 | fatty acid omega-hydroxylase; Provisional | 179–620 | 3.59e-31 |
| PLN02302 | PLN02302 | ent-kaurenoic acid oxidase | 381–579 | 1.91e-27 |
| PLN02183 | PLN02183 | ferulate 5-hydroxylase | 394–594 | 1.27e-24 |
| PLN00110 | PLN00110 | flavonoid 3′,5′-hydroxylase (F3′5′H); Provisional | 382–594 | 1.17e-23 |
| PLN02655 | PLN02655 | ent-kaurene oxidase | 370–623 | 1.76e-23 |
| PLN02426 | PLN02426 | cytochrome P450, family 94, subfamily C protein | 387–620 | 1.04e-22 |
| PLN02687 | PLN02687 | flavonoid 3′-monooxygenase | 326–621 | 1.89e-22 |
| PLN02394 | PLN02394 | trans-cinnamate 4-monooxygenase | 399–589 | 3.07e-22 |
| PTZ00404 | PTZ00404 | cytochrome P450; Provisional | 139–597 | 1.17e-21 |
| PLN03112 | PLN03112 | cytochrome P450 family protein; Provisional | 359–624 | 1.27e-21 |
| PLN03234 | PLN03234 | cytochrome P450 83B1; Provisional | 129–615 | 3.95e-21 |
| PLN02169 | PLN02169 | fatty acid (omega-1)-hydroxylase/midchain alkane hydroxylase | 179–621 | 1.62e-19 |
| PLN02966 | PLN02966 | cytochrome P450 83A1 | 372–624 | 2.70e-18 |
| PLN02987 | PLN02987 | Cytochrome P450, family 90, subfamily A | 382–598 | 3.61e-18 |
| PLN02500 | PLN02500 | cytochrome P450 90B1 | 380–597 | 2.47e-17 |
| PLN02196 | PLN02196 | abscisic acid 8'-hydroxylase | 358–620 | 1.48e-16 |
| PLN00168 | PLN00168 | Cytochrome P450; Provisional | 145–591 | 5.93e-16 |
| PLN02774 | PLN02774 | brassinosteroid-6-oxidase | 380–588 | 1.93e-12 |
| PLN03018 | PLN03018 | homomethionine N-hydroxylase | 386–588 | 4.15e-11 |
| PLN03141 | PLN03141 | 3-epi-6-deoxocathasterone 23-monooxygenase; Provisional | 386–622 | 1.97e-10 |
| PLN02971 | PLN02971 | tryptophan *N*-hydroxylase | 386–624 | 1.99e-10 |
| PLN02738 | PLN02738 | carotene beta-ring hydroxylase | 135–633 | 2.48e-167 |

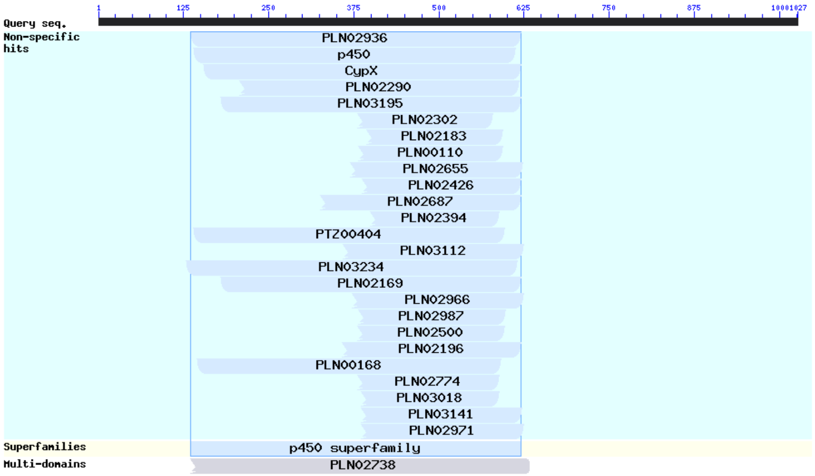


**Figure S37.** Conserved domains detected in a putative abietadienol/abietadienal oxidase gene from *L. dendroidea* (edited from the NCBI CDD database, [1]).

**Table S37.** List of domain hits for the abietadienol/abietadienal oxidase gene from *Pinus taeda* (sp|Q50EK6] C72B1_PINTA).

| Name | Accession | Description | Interval | E-Value |
|---|---|---|---|---|
| PLN03141 | PLN03141 | 3-epi-6-deoxocathasterone 23-monooxygenase; Provisional | 45–481 | 4.09e-132 |
| PLN02987 | PLN02987 | Cytochrome P450, family 90, subfamily A | 5–481 | 1.47e-130 |
| PLN02500 | PLN02500 | cytochrome P450 90B1 | 1–477 | 9.23e-117 |
| PLN02774 | PLN02774 | brassinosteroid-6-oxidase | 5–477 | 5.51e-104 |
| PLN02302 | PLN02302 | ent-kaurenoic acid oxidase | 5–477 | 6.93e-82 |
| PLN02196 | PLN02196 | abscisic acid 8′-hydroxylase | 3–477 | 9.81e-71 |
| p450 | pfam00067 | Cytochrome P450; Cytochrome P450s are haem-thiolate proteins involved in the oxidative ... | 46–454 | 7.80e-49 |
| CypX | COG2124 | Cytochrome P450 [Secondary metabolites biosynthesis, transport, and catabolism] | 60–477 | 3.30e-46 |
| PTZ00404 | PTZ00404 | cytochrome P450; Provisional | 18–452 | 1.33e-17 |
| PLN02936 | PLN02936 | epsilon-ring hydroxylase | 283–459 | 3.81e-17 |
| PLN02183 | PLN02183 | ferulate 5-hydroxylase | 46–454 | 8.12e-17 |
| PLN02290 | PLN02290 | cytokinin trans-hydroxylase | 229–460 | 1.01e-15 |
| PLN02687 | PLN02687 | flavonoid 3′-monooxygenase | 248–458 | 5.51e-14 |
| PLN03112 | PLN03112 | cytochrome P450 family protein; Provisional | 8–447 | 8.67e-13 |
| PLN02966 | PLN02966 | cytochrome P450 83A1 | 39–436 | 1.26e-11 |
| PLN00168 | PLN00168 | Cytochrome P450; Provisional | 239–459 | 6.04e-09 |
| PLN02426 | PLN02426 | cytochrome P450, family 94, subfamily C protein | 251–481 | 1.59e-08 |
| PLN03234 | PLN03234 | cytochrome P450 83B1; Provisional | 265–453 | 2.81e-08 |
| PLN03195 | PLN03195 | fatty acid omega-hydroxylase; Provisional | 3–480 | 1.77e-07 |
| PLN00110 | PLN00110 | flavonoid 3′,5′-hydroxylase (F3′5′H); Provisional | 254–454 | 4.65e-07 |
| PLN02655 | PLN02655 | ent-kaurene oxidase | 330–453 | 5.28e-06 |
| PLN02971 | PLN02971 | tryptophan *N*-hydroxylase | 32–459 | 6.10e-06 |
| PLN02169 | PLN02169 | fatty acid (omega-1)-hydroxylase/midchain alkane hydroxylase | 264–442 | 1.52e-05 |
| PLN02394 | PLN02394 | trans-cinnamate 4-monooxygenase | 279–451 | 2.37e-05 |
| PLN03018 | PLN03018 | homomethionine *N*-hydroxylase | 4–100 | 2.90e-04 |
| PLN02738 | PLN02738 | carotene beta-ring hydroxylase | 256–461 | 4.83e-23 |

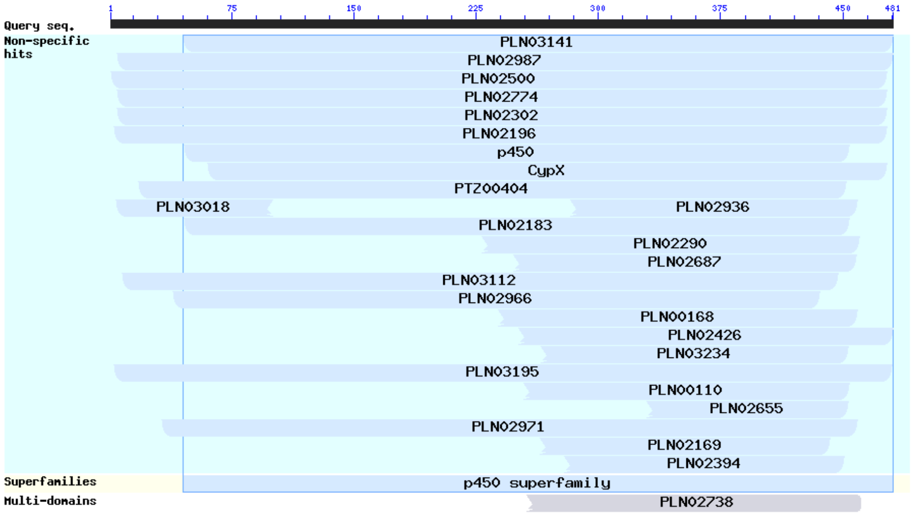


**Figure S38.** Conserved domains detected in the abietadienol/abietadienal oxidase gene from *Pinus taeda* (sp|Q50EK6] C72B1_PINTA) (edited from the NCBI CDD database, [1]).

**References**


1. Conserved Domain Database (CDD). Available online: http://www.ncbi.nlm.nih.gov/Structure/cdd/wrpsb.cgi (accessed on 15 September 2014).
2. Edgar, R.C. MUSCLE: Multiple sequence alignment with high accuracy and high throughput. *Nucleic Acids Res.* **2004**, *32*, 1792–1797.
3. Waterhouse, A.M.; Procter, J.B.; Martin, D.M.A.; Clamp, M.; Barton, G.J. Jalview Version 2—A multiple sequence alignment editor and analysis workbench. *Bioinformatics* **2009**, *25*, 1189–1191.


© 2015 by the authors; licensee MDPI, Basel, Switzerland. This article is an open access article distributed under the terms and conditions of the Creative Commons Attribution license (http://creativecommons.org/licenses/by/4.0/).
